# Surface modifications of titanium dental implants with strontium eucommia ulmoides to enhance osseointegration and suppress inflammation

**DOI:** 10.1186/s40824-023-00361-2

**Published:** 2023-03-16

**Authors:** Avery Rui Sun, Qili Sun, Yansong Wang, Liqiu Hu, Yutong Wu, Fenbo Ma, Jiayi Liu, Xiangchao Pang, Bin Tang

**Affiliations:** 1 Department of Biomedical Engineering, Southern University of Science and Technology, 518055 Shenzhen, China; 2 Guangdong Provincial Key Laboratory of Cell Microenvironment and Disease Research, Guangdong, China; 3 Shenzhen Key Laboratory of Cell Microenvironment, Shenzhen, China; 4 Department of Biomedical Engineering, College of Design and Engineering, National University of Singapore, 117583 Singapore, Singapore; 5 Mechanobiology Institute (MBI), National University of Singapore, 117411 Singapore, Singapore; 6 College of Materials Science and Engineering, Central South University of Forestry and Technology, 410004 Changsha, China

**Keywords:** Titanium dental implant, Biological interfaces, Strontium Eucommia Ulmoides polysaccharides, Polydopamine, Anti-inflammation, Osseointegration, Osteoimmunomodulation

## Abstract

**Background:**

Titanium (Ti) is now widely used as implant material due to its excellent mechanical properties and superior biocompatibilities, while its inert bioactivities might lead to insufficient osseointegration, and limit its performance in dental applications.

**Methods:**

We introduced a robust and simple approach of modifying titanium surfaces with polysaccharide complexes. Titanium samples were subjected to hydrothermal treatment to create a uniform porous structure on the surface, followed by coating with a bioinspired and self-assembly polydopamine layer. Strontium *Eucommia Ulmoides* Polysaccharide (EUP-Sr) complexes are then introduced to the polydopamine-coated porous titanium. Multiple morphological and physiochemical characterizations are employed for material evaluation, while cell proliferation and gene expression tests using macrophages, primary alveolar bone osteoblasts, and vascular endothelial cells are used to provide an overall insight into the functions of the product. The significances of statistical differences were analyzed using student’s t-test.

**Results:**

Microscopic and spectrometric characterizations confirmed that the Ti surface formed a porous structure with an adequate amount of EUP-Sr loading. The attachment was attributed to hydrogen bonding between the ubiquitous glycosidic linkage of the polysaccharide complex and the ring structure of polydopamine, yet the loaded EUP-Sr complex can be gradually released, consequently benefiting the neighboring microenvironment. Cell experiments showed no cytotoxicity of the material, and the product showed promising anti-inflammation, osseointegration, and angiogenesis properties, which were further confirmed by *in vivo* evaluations.

**Conclusion:**

We believe the EUP-Sr modified titanium implant is a promising candidate to be used in dental applications with notable osteoimmunomodulation and angiogenesis functions. And the novel technique proposed in this study would benefit the modification of metal/inorganic surfaces with polysaccharides for future research.

**Supplementary Information:**

The online version contains supplementary material available at 10.1186/s40824-023-00361-2.

## Introduction

Teeth are often impaired by major traumas, external microbes, and inherited disorders. As a necessary part of teeth replacement, titanium implants have been regarded by a considerably large number of dentists and researchers as a quite mature selection in dentistry with high reliability. But in dental surgery, traumas caused by the implantation procedure often induce inflammation in surrounding tissues. Moreover, recent studies have proven that titanium implants might induce inflammation in surrounding tissues over time, causing local and systemic health issues [1]. The titanium implant itself is not capable of dealing with the circumstance, thus, suppressing inflammation around the implants and improving osseointegration are considered as two key factors to achieve the clinical success of dental implantation [2–4]. Thus, developing novel dental implant surface modifications and coatings with enhanced osseointegration and alleviated inflammatory response would further improve the satisfaction and popularity of titanium implants.

The surface modification of titanium focused on improving the biocompatibility and durability of the material where there have been multiple studies investigating the method to create covalent bonds between titanium and the coating material via multiple complicated chemical reactions [5–7]. Except for the strictly demanded reaction condition, many of the reactants are highly toxic and volatile. Polydopamine (PDA) is a bioinspired material originating from the secreted adhesive protein of mussels, which enables them to attach to a wide spectrum of surfaces [8–10]. PDA has consequently been widely recognized as a medium agent to modify multiple types of surfaces due to its mild reaction condition and wide usage [8, 11].

Surface treatment by natural or modified polysaccharides is a promising means to fight against implant-associated problems [12]. Many types of polysaccharides have been used as coating materials for implants due to their immune-regulatory activities originating from the binding of their sugar units to specific carbohydrate receptors on macrophage surfaces [13, 14]. It was proven that PDA-modified surfaces can be further coated with a secondary drug/chemical by creating non-covalent linkages including hydrogen bonds, π stacking, and van der Waals interaction [15]. Thus, it can be speculated that the ubiquitous existence, as well as the hydrogen-bond-availability of glycosidic linkages in various types of polysaccharide complexes, enables the introduction of a secondary polysaccharide coating onto the PDA layer. In this study, we have developed a sustainable method to attach polysaccharide or their complexes onto titanium implants with mild reaction conditions and non-toxic reactants based on PDA attachment. Originated from herbal medicine, *Eucommia Ulmoides* polysaccharides (EUP) is a well-characterized carbohydrate, whose function in regulating the immune system has been revealed [16]. Combined with strontium acting as an osteoblastogenesis initiator, strontium *Eucommia Ulmoides* polysaccharides (EUP-Sr) were synthesized and investigated in a previous work. The polysaccharide complex demonstrated no cytotoxicity in the cell proliferation test, while significantly inhibited multiple proinflammatory factors in the gene expression of macrophages. It was also proven that EUP-Sr could potentially induce bone regeneration by upregulating osteoblastogenesis gene expression while suppressing osteoclastogenesis [17]. In this study, we introduce the EUP-Sr to the titanium surface, with the purpose to endow titanium dental implants with anti-inflammatory functions and improved osseointegration activities.

In this study, we hypothesize that (i) the PDA coating method suggested in this study, which is feasible for a wide genre of polysaccharide complex, is an economic and facile surface modification method to improve the biophysical and biochemical properties of titanium surface by the coating of polysaccharide complex; and (ii) the introduction of EUP-Sr on titanium surface possesses the capability to significantly increase the anti-inflammation, angiogenic and osteogenic of titanium, leading to better osseointegration effects. Systematic materials characterizations were performed to verify the successful attachment of EUP-Sr on the titanium surface. Various *in vitro* biological studies were performed to evaluate the cytocompatibility, cell inflammatory response, angiogenic and osteogenic performances of EUP-Sr modified titanium. *In vivo* assays confirmed that the EUP-Sr loaded onto the implant could endure surgical manipulations when the surface was rubbed with the alveolar bone, while the polysaccharide complex can actively and consistently benefit the neighboring microenvironment in the recovery process. With this novel and pervasive approach, titanium implants could be loaded with various polysaccharide complexes according to specific clinical needs including anti-inflammation and osteoinduction. It is hopeful that our proposal could potentially spark a way to suppress the inflammatory responses of the patient post dental implantation, as well as reduce the recovery time.

## Materials and methods

### Materials

Titanium (purity > 99.5%) was purchased from Taizhou Zenno Material Technology Co., Ltd. The clinically-available Ti implants (series: HE 1.5 mm) used in the *in vivo* study were purchased from Shanghai Shuangshen Medical Instrument Co., Ltd. Dopamine (AR) was purchased from Aladdin (China). EUP was bought from Xi’an Haochen Biological Technology Co., Ltd. NaHCO_3_ (AR) was purchased from Shanghai Lingfeng Chemical Reagents Co., Ltd. NaOH and SrCl_2_ (AR) were purchased from Aladdin (China). All reactants were used as received unless specified otherwise. Deionized (DI) water (> 18.2 MΩ·cm) was used when water is involved.

### Hydrothermal alkaline treatment

Ti was cut to slices (1 mm thick), then progressively polished with 240^#^, 600^#^, 800^#^, 1000^#^, and 2000^#^ waterproof abrasive paper. After polishing by diamond powder paste, the titanium slices were ultrasonically cleaned using ethanol, acetone, and water before surface modification. Supplemental Fig. 1 shows the sketch to modify Ti surface with EUP-Sr. Nano-sized porous structure was introduced onto the Ti surface via hydrothermal alkaline treatment [18], in which the cleaned Ti slices were immersed in 10 M NaOH solution and kept at 140^o^C for 2 h. After being cooled to room temperature (*RT*, 23-26 ^o^C), the treated Ti slices were washed with extensively NaHCO_3_ solution (5%) and kept in NaHCO_3_ solution for at least 12 h to fully neutralize the possible residual alkaline in the pores on the surface. Lastly, to remove the salt residue, the Ti slices were boiled in water for 1 h and rinsed 3 times with water.

### PDA and EUP-Sr layer attachment

An aqueous solution of dopamine was used to attach PDA onto the Ti surface according to a previous study: the dopamine was dissolved in Tris buffer for a concentration of 2 mg/mL (pH = 8.5). The treated Ti slices were then immediately immersed in the dopamine solution for 24 h to ensure it reaches the maximum thickness [8]. The Ti-PDA slices were then rinsed to remove unattached residues. Then, EUP-Sr prepared according to the previous study [17] was dissolved in 10 mM Tris-HCl buffer to obtain EUP-Sr saturated solution. The Ti-PDA slices were subsequently soaked in the solution for 12 and 24 h respectively to obtain samples attached with different amounts of EUP-Sr. The EUP-Sr was believed to be coated on the Ti surface after the soaking, becoming the eventual product named Ti-EUP-Sr hereafter.

### Characterization of Ti-EUP-Sr

#### SEM and SEM/EDS

Scanning electron microscopy (SEM, JSM-7200 F) with an accelerating voltage of 10.0 kV was used to observe the surface morphology of different samples (Ti post hydrothermal alkaline treatment, Ti-PDA, and Ti-EUP-Sr), while the energy dispersive spectrometer (EDS) was applied to measure the composition of Sr element on the sample surfaces. For each group, 3 randomly selected locations were detected in EDS measurement. To directly confirm and visualize the EUP-Sr coating, the sample was cut and the cross-sectional surface was observed with SEM. All samples mentioned were sputtered with gold before observation and examination.

#### ATR-FTIR

Attenuated total reflectance-Fourier transform infrared spectroscopy (ATR-FTIR, PerkinElmer) was employed to validate the presence of functional groups in EUP-Sr, Ti-PDA, Ti-EUP-Sr-12 H, and Ti-EUP-Sr-24 H in the spectral range of 4000 to 400 cm^− 1^. By ATR-FTIR analysis, the underlying mechanism for the attachment of the polysaccharide complex would be revealed.

#### Inductively coupled plasma-optical emission spectrometry (ICP-OES)

The samples Ti-EUP-Sr-12 H and Ti-EUP-Sr-24 H were immersed in 15 mL saline, then incubated and shook at 37 ^o^C to mimic the *in vivo* environment. In the time range of 0 to 72 h, 500 µL of the solution was sampled at the interval of 0.5 h upon immersion for two of the samples, followed by adding 500 µL saline back to the release system per sampling. The samples were respectively added with nitric acid (SP) (v/v = 1/4) and kept at 180  ^o^C for 20 min. Then the nitrified sample was diluted to 5 mL with water. The samples were therewith examined using the inductively coupled plasma optical emission spectrometer (ICP-OES, PerkinElmer).

#### Hydrophilicity

The surface hydrophilicity of the different substrates (Ti, Ti-PDA, Ti-EUP-Sr-12 H, and Ti-EUP-Sr-24 H) was analyzed on a contact angle system (AST Products, Inc.). Three samples from each group were selected in the measurement with 5 points on each sample were selected (center and four points near four angles).

### Cell experiments

#### Primary culture of human alveolar bone osteoblasts (HABOBs)

HABOBs, the osteoblasts dwelling in the alveolar bone that respond to regulating the formation and regeneration of alveolar bone, are harvested from the disposed tissue of teeth after tooth extraction surgery (orthodontic). The experimental protocol was ethically reviewed by Guangzhou University of Chinese Medicine-Shenzhen Hospital (File # F-GZSY-LL-GZCX-14-2-1). To keep the vitality of the cells, the tissue should be immediately transferred to the primary culture after extraction. A thin layer of pre-warmed fetal bovine serum (FBS, Gibco) was added to a culture flask. The tissue was cut into small fragments (approximately 1 mm^3^) and placed in the culture flask at around 5 mm intervals. After being kept still in the incubator for 1 h, the tissue fragments started to adhere to the flask. The complete culture medium used for HABOBs was HyClone Dulbecco’s Modified Eagle Medium (DMEM) supplemented with 15% FBS and 2% Penicillin-Streptomycin at primary culture. Before continue to incubate, the complete culture medium was gently and carefully added into the flask to immerse the tissue fragments once the fragments were attached to the flask relatively tight. After 2 weeks of culture, osteoblasts-like cells were observed adjacent to the tissue fragment. Then, the medium was changed every 2–3 days hereafter using HyClone Dulbecco’s Modified Eagle Medium (DMEM) supplemented with 15% FBS. Once the cells covered more than 75% of the flask, subculture was performed. The primary cells used in this study were within passage 4.

#### Cell proliferation

The modified Ti samples were prepared according to the size of the 96-well plate in advance. RAW264.7 cells (macrophages) were plated with a concentration of 2000/well onto the samples that were previously sterilized and placed into the 96-well plates, while Human Umbilical Vein Endothelial Cells (HUVECs) and HABOBs were 1000/well. The culture medium for HUVECs and HABOBs was DMEM supplemented with 15% FBS, while the medium for RAW264.7 was DMEM supplemented with 5% FBS. The cells were incubated at 37 ^o^C in 5% CO_2_ for 1, 3 and 5 days before performing the MTS assay. Each group was performed in 5 replicate wells. Then a microplate reader (iMark, Bio-Rad) was used to measure the absorbance at 490 nm.

#### Real-time qPCR

RAW264.7 cells and HUVECs were cultured on the sterilized samples placed in 6-well plates for 24 or 72 h, while HABOBs were cultured for 3 or 7 days before they were cleaved using TRIZOL. Chloroform was later utilized to remove redundant substances other than RNA from water. After being precipitated and rinsed with 75% ethanol (DEPC treated water solution), the RNA was resuspended in DEPC treated water and the concentration was measured on NanoDrop 2000 tester. The total RNA was then reverse-transcribed to complementary DNA (cDNA) using Evo M-MLV RT Premix (Accurate Biotechnology Hunan Co., Ltd.) at 37 ^o^C for 15 min, 85 ^o^C for 5 s, and kept at 4 ^o^C. The resultant cDNA samples were analyzed using SYBR Green Pro Taq HS qPCR Kit (Accurate Biotechnology Hunan Co., Ltd.) following the instructions provided by the supplier. The targeted genes were normalized against GAPDH (RAW264.7: mouse GAPDH; HUVECs & HABOBs: human GAPDH) with reference to the cells cultured with no Ti-EUP-Sr samples (control). The target genes of RAW264.7 tested were: interleukin-10 (IL-10), tumor necrosis factor alpha (TNF-α), and Inducible Nitric Oxide Synthase (iNOS). The target genes of HUVECs were Vascular Endothelial Growth Factor A (VEGFA) and Epidermal Growth Factor Like-domain 6 (EGFL6). And the target gene tested in HABOBs were Alkaline Phosphotase (ALP), osteopontin (OPN), osteocalcin (OCN), and Runx2. The relative expression of each gene was processed and analyzed with the 2^− ddCt^ method [19].

#### Enzyme-linked immunosorbent assay (ELISA)

The ELISA assay kits used in this study (human VEGF ELISA kit, human OCN ELISA kit, and mouse IL-10 ELISA kit) were bought from Beyotime Biotechnology. Assays were conducted according to supplier’s instructions came with each kit. Briefly, after culturing RAW264.7, HUVECs, and HABOBs for 1, 3, or 7 days on EUP-Sr modified Ti samples, cell supernatant was obtained and diluted before adding 100 µL samples from each group to a 96 well plate. Standard curves (2000, 1000, 500, 250, 125, 62.5 pg/mL) were obtained with every assay proceeded. After adding biotinylated antibodies, streptavidin labeling solution, and TMB solution stepwisely according to the product instruction (5 times of washing are required before adding each solutions mentioned), the well plates were incubated in dark for 20 min and supplemented with 50µL/well termination solution. A450 values were measured using a microplate reader (iMark, Bio-Rad).

#### Data analysis

The significant difference in experiment data was analyzed with Student’s *t*-test. All values involved in the study were denoted as mean ± standard deviation (SD). *p* < 0.05 was regarded as the appearance of a significant difference in the data.

### Animal experiments

The *in vivo* experiment of this study was approved and conducted in Southern University of Science and Technology (Approval#: SCXK2022-0170), where New Zealand rabbits (female, 85–115 days, 2-2.5 kg) were purchased from Guangdong Medical Laboratory Animal Center. The animals were restrained and anesthetized using isoflurane with an increasing concentration gradient, followed by hair removal and iodine sterilization at the surgical area (back right tibia). 2% of lidocaine was used at the surgical area to enhance regional anesthesia followed by further iodine sterilization and a ~ 5 cm longitudinal incision until the upper-middle region of the tibia was exposed. Periosteum and deep muscular tissue were identified and carefully dragged towards the side to expose the tibia surface. Pre-cooled (4 ^o^C) saline was used to rinse the implantation region, and a 0.5 mm round bur was used to create a ~ 0.5 mm × 0.5 mm locating region on the cortical bone. Commercialized Ti implants or EUP-Sr modified Ti implants were then inserted through the locating region while screwing. The surgical region was then closed and the animal was recovered. After euthanasia, implants were removed from the tibiae after bone extraction followed by SEM/EDS analysis of the implantation region. The tibiae of the other animals were extracted for micro-CT imaging (150 kV) and reconstructed using Imaris software.

## Results

### Biomaterial characterization and modification time optimization

#### Morphology of samples

**Fig. 1:**
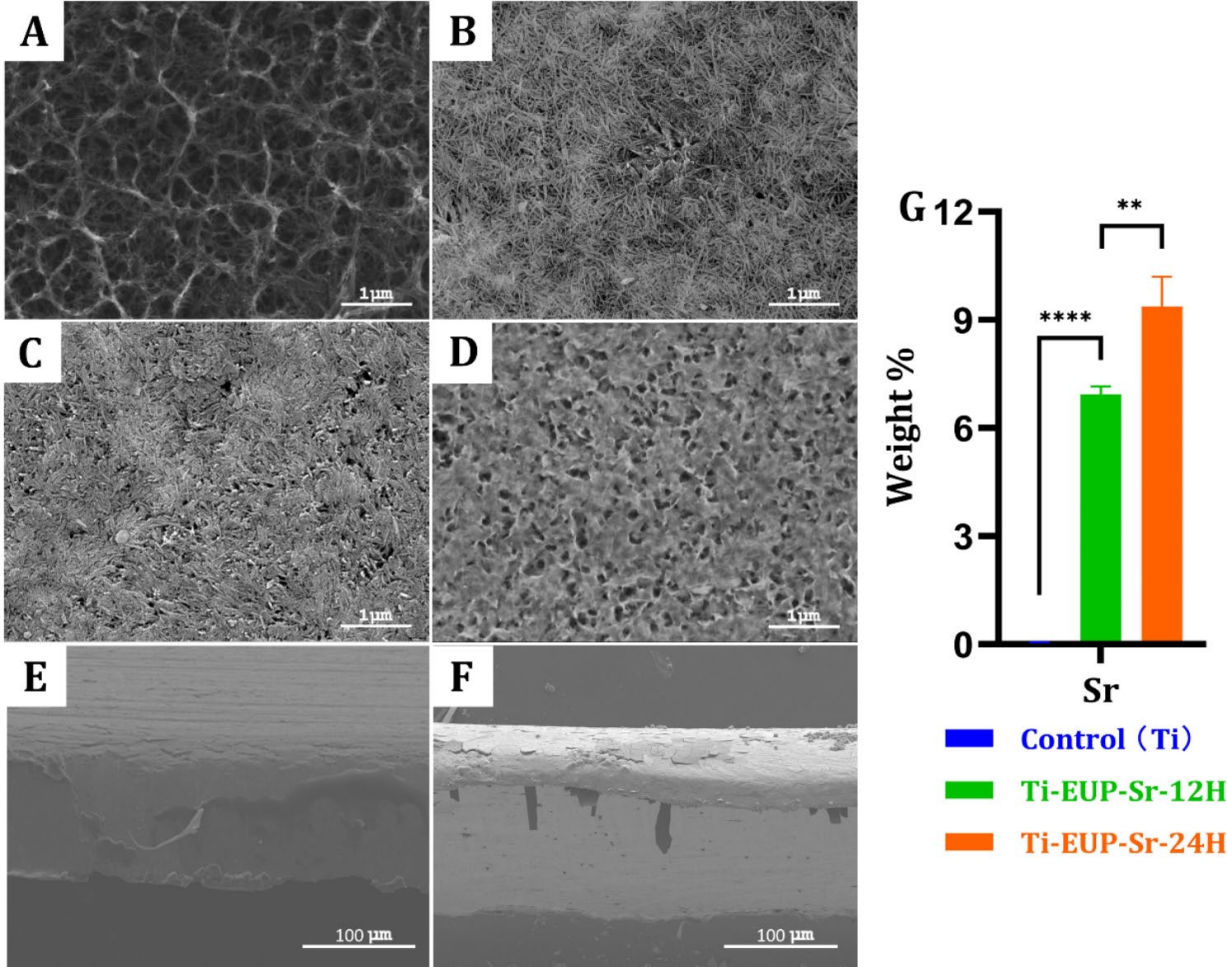
SEM images of hydrothermal alkaline treated Ti (A), Ti-PDA (B), Ti-EUP-Sr-12 H (C), and Ti-EUP-Sr-24 H (D). Cross sectional view of Ti (E) and Ti-EUP-Sr-24 H (F). EDS result of strontium content (G). In Fig. 1(G), values shown are mean ± SD. ^**^p < 0.01 or ^****^p < 0.0001 stands for a significant difference (no Sr signals were detected in the Ti group)

The typical SEM pictures of hydrothermal alkaline treated Ti, Ti-PDA, and Ti-EUP-Sr are shown in Fig. 1. The general features of hydrothermal alkaline treated Ti surface were notably changed after PDA treatment. It could be found that Ti formed a reticulate surface structure after the alkaline treatment (Fig. 1A), while the net structure of Ti-PDA was facilitated for the polydopamine to adhere, making the “strings” of the nets much thicker and the “holes” of the nets much smaller, as shown in Fig. 1B. In Fig. 1C, after 12 h of modification with EUP-Sr on Ti-PDA, an irregular coating layer was formed on the surface of Ti-PDA substrates, which might be the aggregates of EUP-Sr particles. This conclusion can be verified by increasing the modification time. With increasing the modification time by 24 h, for example, the surface became relatively uniform (Fig. 1D). Therefore, 24 h modification should be suitable to establish a uniform coating layer for the immobilization of EUP-Sr. Subsequently, we observed the cross-sectional surface of both Ti (Fig. 1E) and Ti-EUP-Sr (Fig. 1F) with 24 h treatment time using SEM, finding a uniform layer of EUP-Sr coating.

To compare the strontium content on the surface of samples, the EDS tests were carried out on the samples (Fig. 1G). The results indicate that there was no strontium detected on the Ti-PDA surface, while for the other two samples, notable amount of strontium was detected (~ 6.94% for Ti-EUP-Sr-12 H and ~ 9.38% for Ti-EUP-Sr-24 H). This suggested that EUP-Sr was successfully immobilized on the samples, and the content of the immobilized EUP-Sr increases with the immersion time.

#### Composition of the sample

**Fig. 2:**
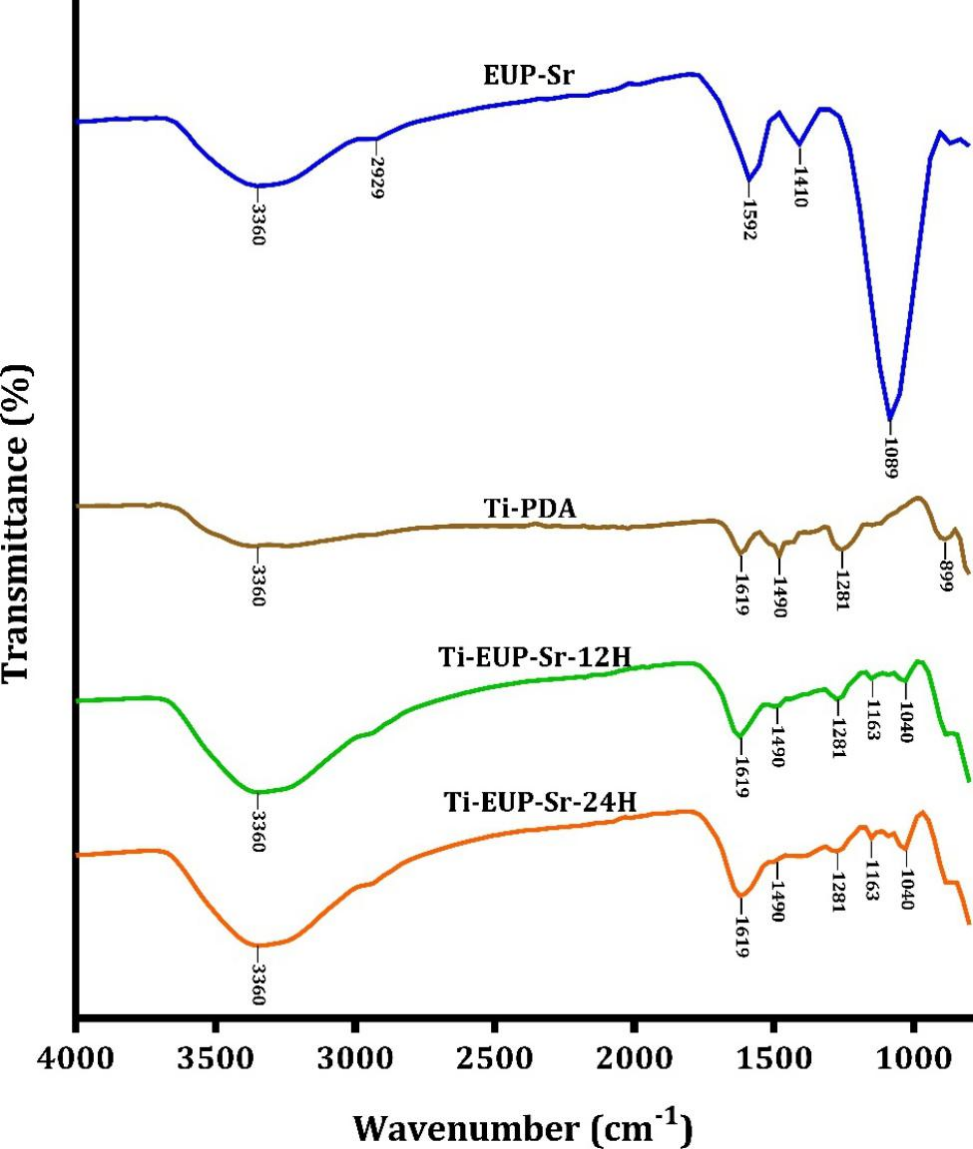
FTIR spectra of EUP-Sr, Ti-PDA, Ti-EUP-Sr-12 H, and Ti-EUP-Sr-24 H.

To further confirm the formation of Ti-EUP-Sr on the surface of samples, Fourier transform infrared spectroscopy (ATR-FTIR) was performed. Figure 2 displays the FTIR spectra of the samples.

The strong and wide absorption peak at 3360 cm^− 1^ can be attributed to the -OH stretching vibration. It was evident that the peak area of Ti-EUP-Sr-12 H and -24 H, comparing with Ti-PDA, increased significantly with the addition of EUP-Sr. The weak absorption peak at 2929 cm^− 1^ resulted from the stretching vibration of the C-H bond [16, 20]. The peaks at 1592 and 1410 cm^− 1^ indicate the symmetric and non-symmetric OCO- stretching vibrations. No absorption peaks were found nearly at 1740 and 1250 cm^− 1,^ confirming the absence of uronic acid and sulfate groups. And the peak at around 1100 cm^− 1^ corresponds to the ring vibrations overlapping with C-OH side groups and the stretching vibration of the glycosidic linkage (C-O-C) vibration. For the other three groups investigating Ti modification, the characteristic peak of N-H bending and stretching vibration was detected at 1619 cm^− 1^ in all three groups [21]. The small peak at 1490 cm^− 1^ was ascribed to the C = C bonds from the indole structure of PDA, which is also an indicator of successful polymerization from dopamine to PDA [21, 22]. The peak at 1281 cm^− 1^ corresponds to the stretching vibration of catechol hydroxyl C-O and/or C-N [23]. Interestingly, Ti-EUP-Sr-12 H and Ti-EUP-Sr-24 H displayed almost the same spectra, indicating modification time exerts no influence on the functional groups in Ti-EUP-Sr. A peak at 1089 cm^− 1^ represented the vibrations of C-O in EUP-Sr [24, 25]. This peak shifted to 1040 cm^− 1^ when EUP-Sr attached to Ti-PDA. This phenomenon can be ascribed to the formation of hydrogen bonding between the glycosidic linkage of EUP-Sr and the ring structure of PDA, which confirmed our initial hypothesis of PDA and the polysaccharide complex can interact through intermolecular forces.

#### Influence on the neighboring microenvironment

**Fig. 3:**
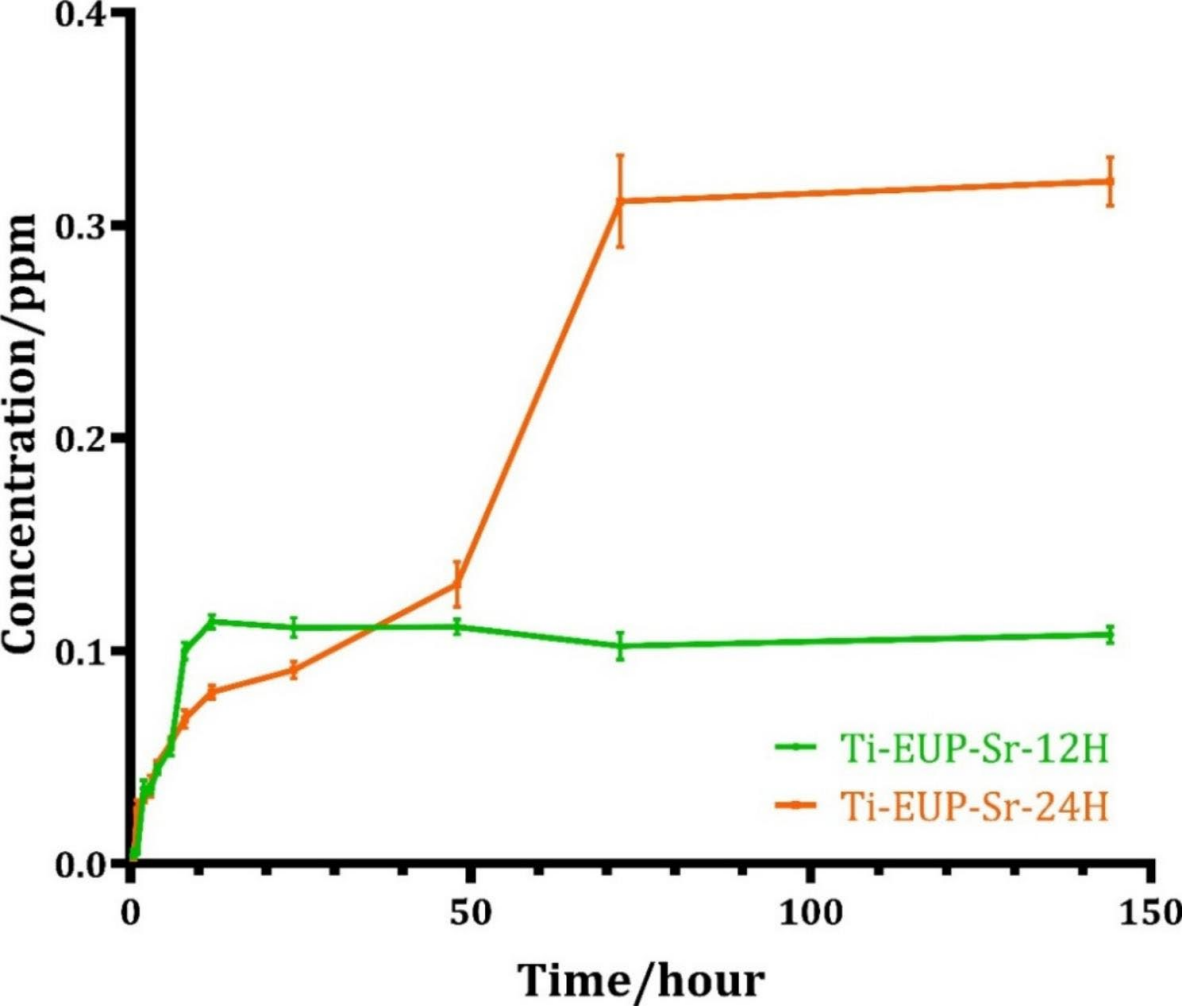
Strontium ion release detected by ICP-OES

The ICP-OES analysis was shown in Fig. 3. Ti-EUP-Sr-24 H group reached equilibrium at around 0.33 ppm after around 72 h of release, while the equilibrium concentration of Ti-EUP-Sr-12 H group was around 0.11 ppm after 12 h of release. Further, the constant release of Sr ions will also be greatly helpful for the implant to exert long-term biological effects.

#### Hydrophilicity of the samples

**Fig. 4:**
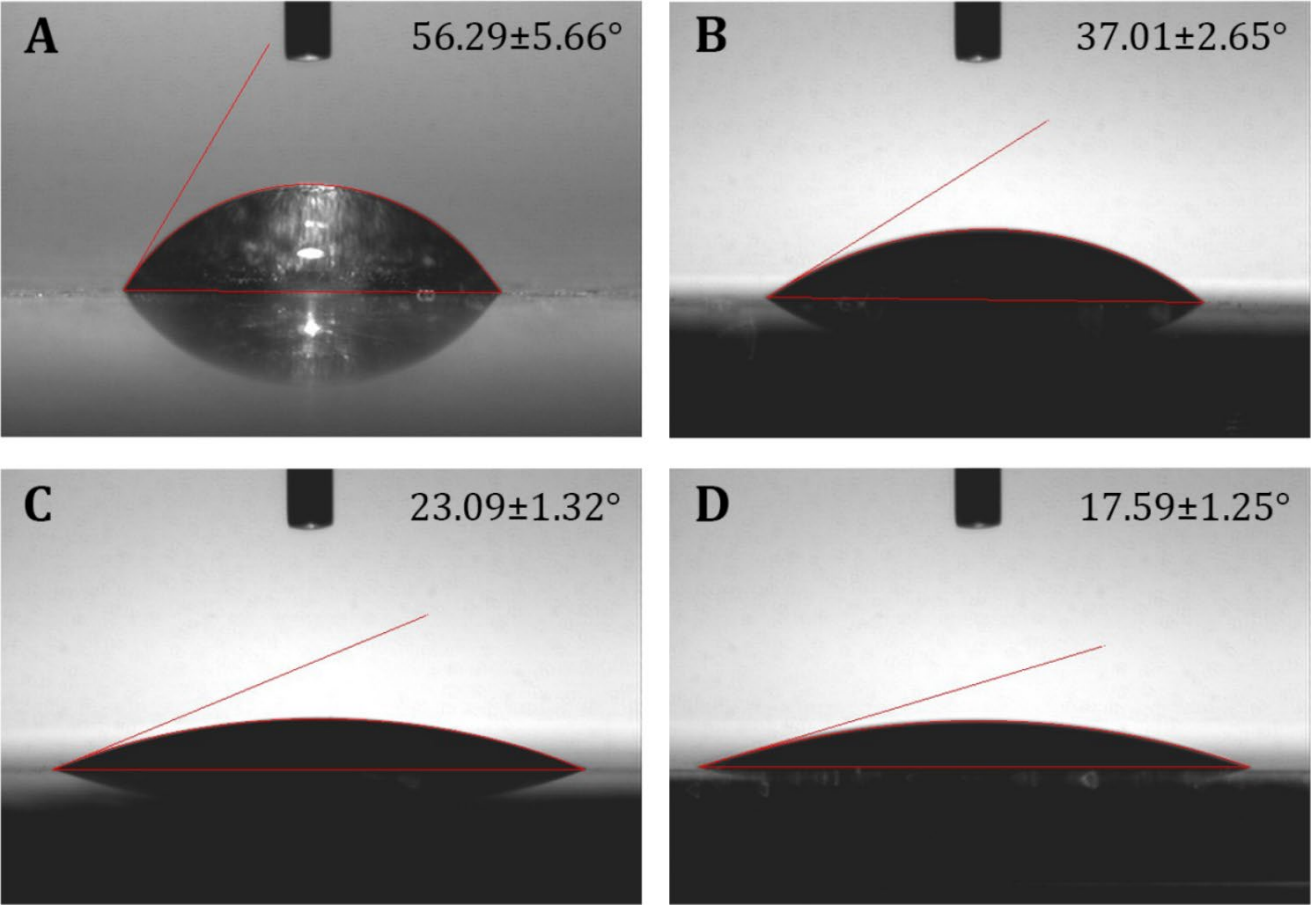
The contact angle of water on Ti (A), Ti-PDA (B), 12 h modified Ti-EUP-Sr (C), and 24 h modified Ti-EUP-Sr (D)

**Fig. 5:**
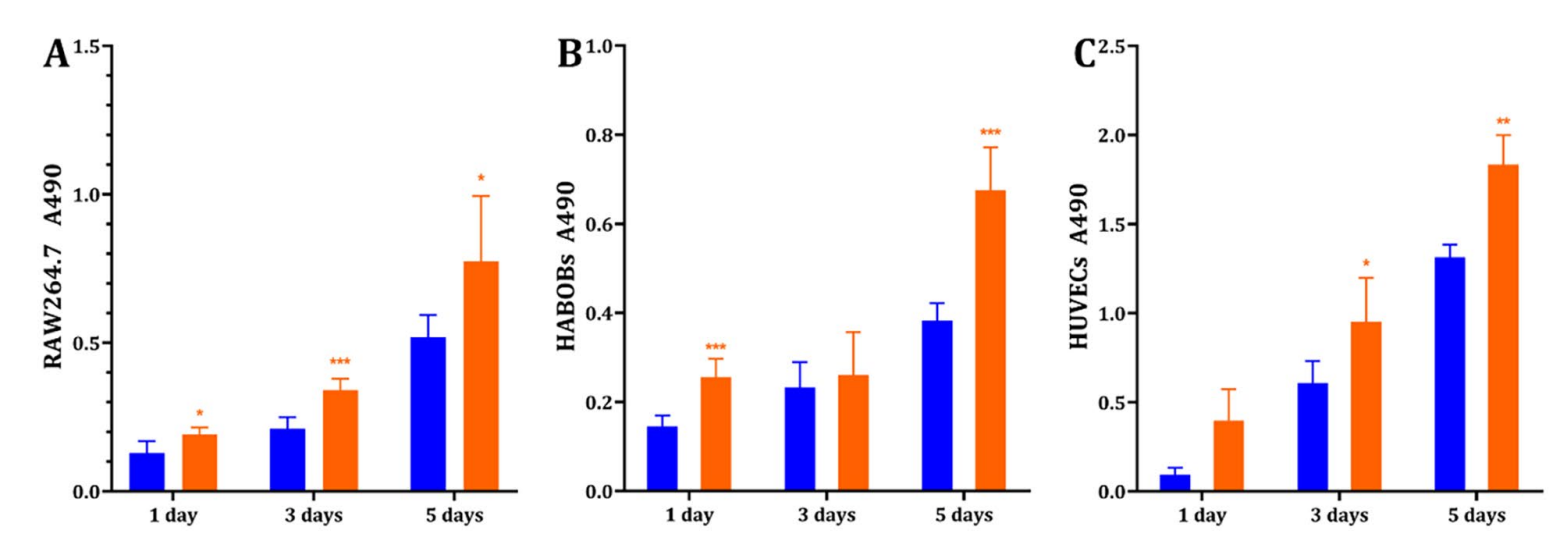
Cell proliferation was analyzed by MTS assay: RAW264.7 (A), HABOBs (B), and HUVECs (C). Values shown are mean ± SD. ^*^p < 0.05 or ^**^p < 0.01 or ^***^p < 0.001 stands for a significant difference compared to the control group

As reported in previous studies, hydrophilic surface will facilitate cell adhesion and spreading [26]. Thus, the contact angle of the samples was investigated (Fig. 4). It can be found that the surface of the modified sample with PDA and EUP-Sr becomes more hydrophilic than that of the bare Ti. Moreover, after modification with EUP-Sr, the water contact angle is smaller than that treating with PDA (Figs. 4C and 5D). The results can be confirmed by our SEM and FTIR results in Figs. 1 and 2, which indicated that Ti-EUP-Sr displayed porous structure and hydroxyl groups were present on the surface, both of them contributing to the increase of wettability [27, 28]. Thus, the improvement of surface hydrophilicity of Ti-EUP-Sr should be attributed to both chemical and physical influences. The increase of hydrophilicity should result in better cell attachment and viability [26].

Based on the former results, the Ti-EUP-Sr-24 H group showed a uniformly porous surface with a higher loading amount of EUP-Sr, which also endows it with a higher hydrophilicity. It could also optimize the neighboring microenvironment by releasing the polysaccharide complex, resulting in a satisfying concentration. Therefore, the 24 h modified Ti-EUP-Sr was selected to be further investigated in the cell experiments hereafter.

### *In vitro* bioactivity of Ti-EUP-Sr

#### The proliferation of cells on Ti-EUP-Sr

To investigate cytocompatibility of the immobilized EUP-Sr, in *vitro* tests were performed using RAW264.7 cells, HABOBs and HUVECs. Each type of cell was seeded on the Ti-EUP-Sr surfaces with a control group using untreated Ti representing traditional titanium dental implants. After 1, 3 and 5 days of cell culture, the proliferation assay was performed. The results shown in Fig. 5 suggest that the proliferation of all three types of cells significantly improved over 5 days of culture on Ti-EUP-Sr substrates. This means that the biocompatibility of Ti can be greatly enhanced by surface functionalization with EUP-Sr.

#### Gene expression analysis

**Fig. 6:**
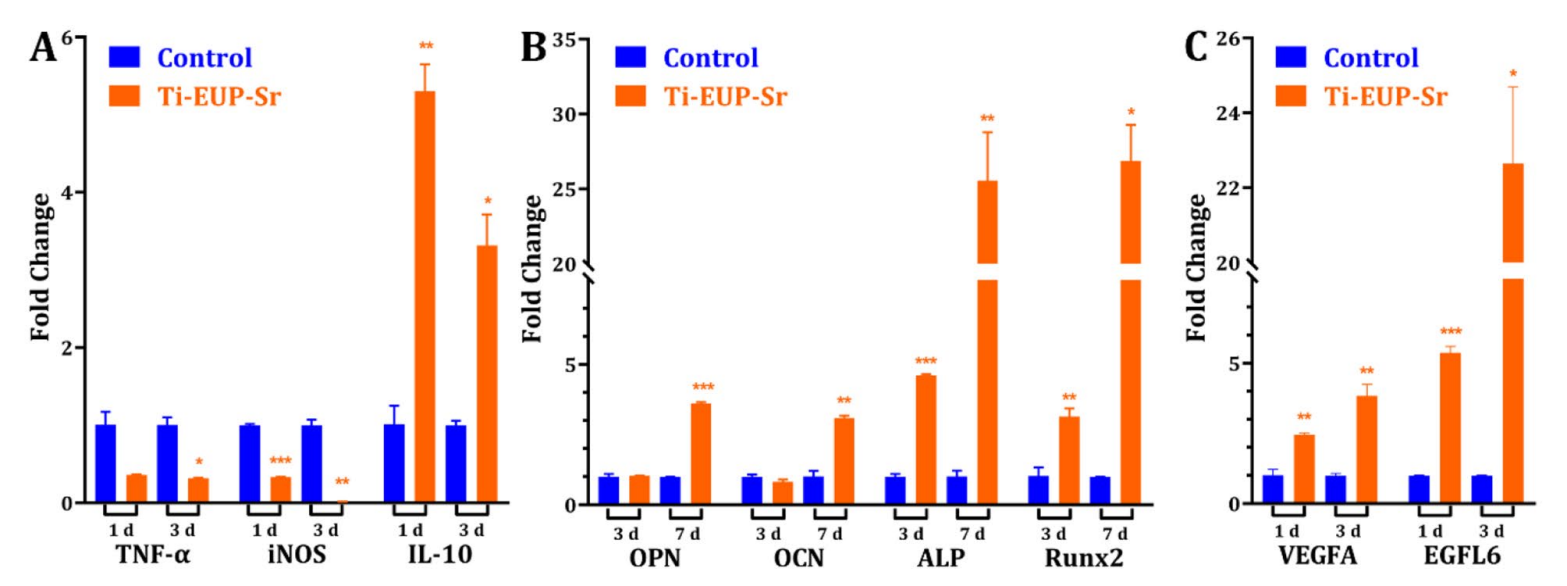
Gene expression of macrophages (A), HABOBs (B), and HUVECs (C). Values shown are mean ± SD. ^*^p < 0.05 or ^**^p < 0.01 or ^***^p < 0.001 stands for a significant difference compared to the control group

To investigate the in *vitro* response of macrophages activated by Ti-EUP-Sr, the expression change of inflammation-suppression gene (IL-10) and inflammation-promotion genes (TNF-α and iNOS) between the control group (untreated Ti were used as control in the qPCR study) and Ti-EUP-Sr groups were analyzed by RT-qPCR and the results were demonstrated in Fig. 6A. It was found that IL-10 for Ti-EUP-Sr group was significantly upregulated, which indicates its beneficial effects on inhibiting the excessive inflammatory response. Moreover, the expression of the pro-inflammation cytokines TNF-α and iNOS was significantly decreased. Thus, Ti-EUP-Sr displayed positive effects on suppressing the inflammatory response.

We further tested the osteogenic-related gene expression levels of ALP, OPN, OCN, and Runx2 after culturing HABOBs on Ti-EUP-Sr. As the RT-PCR results suggested (Fig. 6B), the expression levels of ALP, OPN, OCN and Runx2 of HABOBs in Ti-EUP-Sr group were significantly up-regulated relative to that of in control group after 7 days of culture. Additionally, we performed a mineralization assay to further confirm the osteogenic functions of Ti-EUP-Sr (Supplementary Materials S2). These results demonstrate that Ti-EUP-Sr should facilitate the improvement of the osteogenic performance of HABOBs.

Angiogenesis is another important factor in evaluating a biomaterial substitute, thus, the gene expressions of HUVECs were investigated focusing on vascular endothelial growth factor A (VEGFA) and EGF-like domain multiple 6 (EGFL6). As shown in Fig. 6C, significant increases of all the three gene expression levels could be found in culturing HUVECs on Ti-EUP-Sr, indicating a promising angiogenesis function.

#### ELISA

**Fig. 7:**
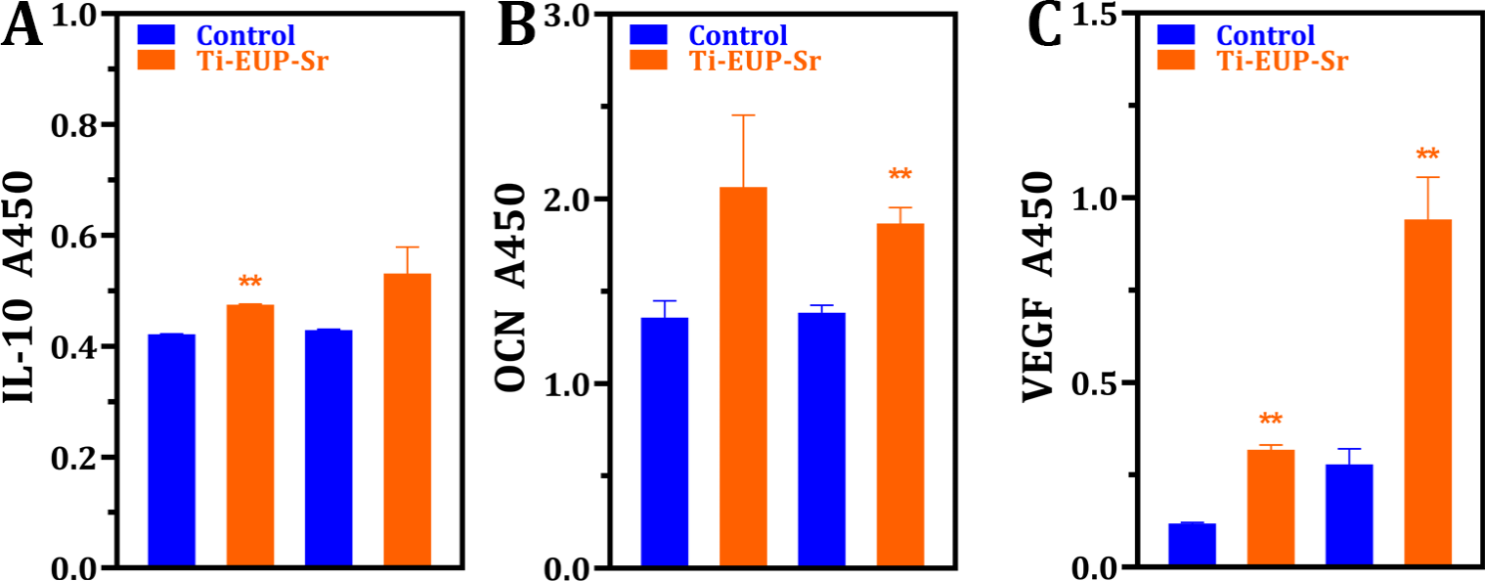
ELISA results confirm increased angiogenesis and osteo-immunoregulatory function (first two columns: after 24 h of culture; second two columns: after 72 h of culture).

To provide a down-stream evaluation of gene expressions of the mentioned cells in response to EUP-Sr loaded titanium implants, ELISA targeting at vascular endothelial growth factor (VEGF), OCN, and IL-10 were conducted using HUVECs, HABOBs, and RAW264.7 cells, respectively. As shown in Fig. 7, the downstream expressions of IL-10 in RAW264.7 cells and VEGF in HUVECs were both immediately upregulated within only 24 h of culture. After 3 days, the OCN expression in HABOBs and VEGF expression in HUVECs showed a significant increase. These results further confirmed the osteo-immunoregulatory functions of Ti-EUP-Sr, as well as its promising angiogenetic inducement.

### *In vivo* evaluations of Ti-EUP-Sr

**Fig. 8:**
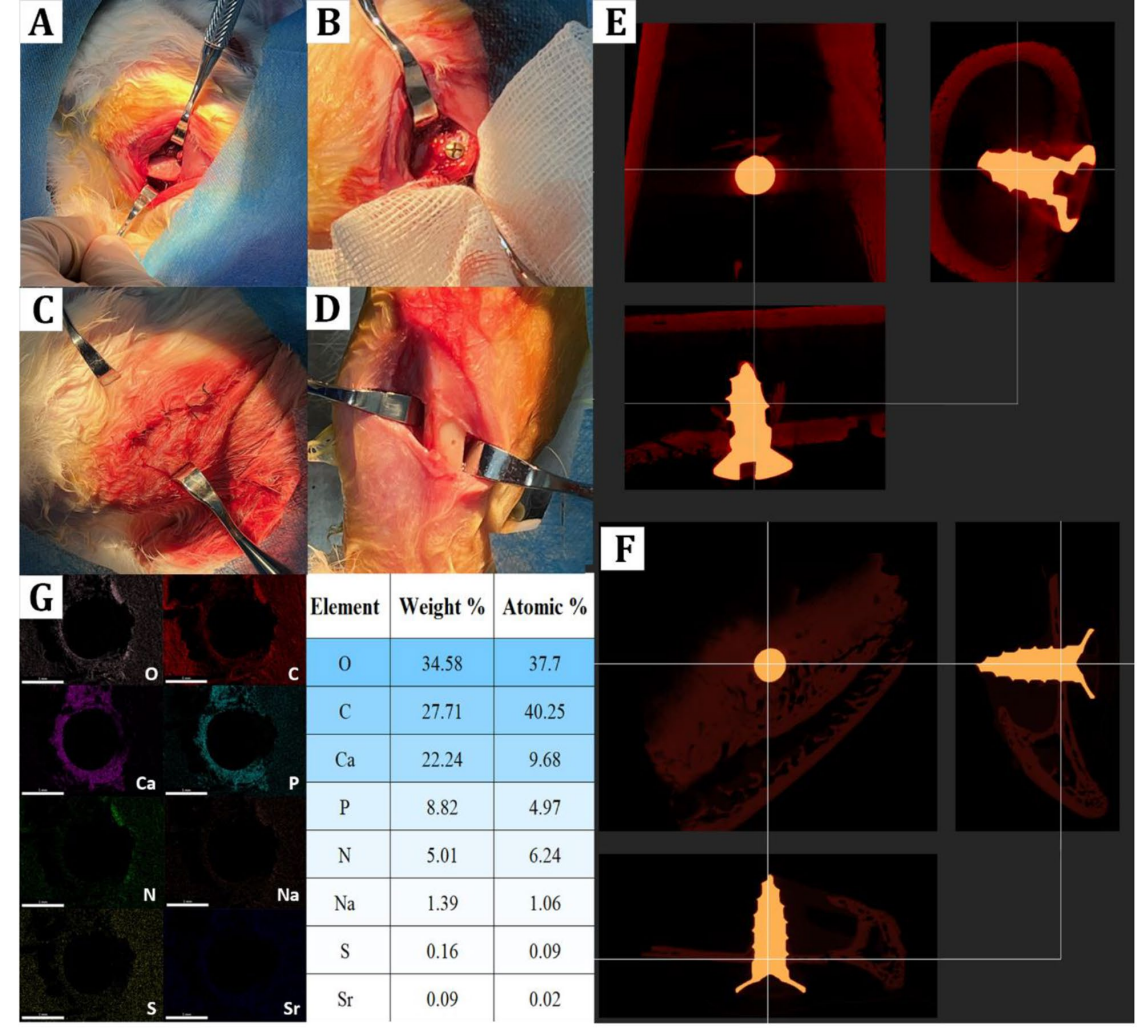
The key surgical procedures and results for *in vivo* experiments. Tibia exposure (A), implantation (B), closure and recover (C), and sample extraction post-euthanasia (D). Micro-CT reconstruction demonstrated satisfying osteointegration of the implant (control: E; Ti-EUP-Sr: F), while the SEM/EDS results confirmed the coating stability with negligible strontium detected around the surgical area (G)

After the implantation surgery, we (1) immediately sacrificed the animal, harvest the surgical region of the tibia, and removed the inserted implants for SEM/EDX tests, and also (2) performed micro-CT on the experimental animal to confirm the osseointegration. Figure 8 A-D shows the key surgical procedures including tibia exposure, implantation, suturing, sample extraction after euthanasia. From a sample image of the micro-CT reconstruction shown as Fig. 8E/F (re-slicing animation of the entire sample can be seen at Supplementary Materials S3_CT_Control and Supplementary Materials S4_CT_Ti-EUP-Sr), the implants also demonstrated satisfying osseointegration after surgery. In Fig. 8G, negligible amount of strontium was detected around the implantation region, indicating that the EUP-Sr coating remained stable even after the mechanical disturbance during the surgical rubbing between the implant surface and tibia.

## Discussion

Due to the superior biocompatibility and beneficial bioactivities, polysaccharides and their complexes were intensively investigated for several decades. To the best of our knowledge, it is the first attempt that to introduce polysaccharides on an inert metal surface via biofriendly methods. The presented results suggested that surface coating with bioactive polysaccharides is effective in improving the bioactivity of inert metals, and therefore, further exploring their biomedical applications. Moreover, the use of ATR-FTIR in this research has revealed the underlying mechanism of attaching EUP-Sr onto the PDA film (described in Sect. 3.1.2). As is mentioned, the linkage was via the hydrogen bond formation between the glycosidic linkage of EUP-Sr and the ring structure of PDA, it becomes deducible that our proposed modification approach is valid for other types of carbohydrate polymers (or their modified complexes) with glycosidic linkages. Recently, the field of investigating and applying carbohydrates as medicine has been constantly thriving, with many traditional herbs characterized for their functional content (usually polysaccharides). For example, Astragalus polysaccharide (APS) extracted and purified from *Astragalus membranaceus* has been well evaluated for pharmacological utilizations including immunomodulatory, angiogenetic, and antitumor functions [29–31]. The method proposed in this study is therefore believed of notable referential values for future studies in introducing organic compounds to PDA-favored surfaces including noble metals (Au, Ag, Pt, etc.), metallic oxides (TiO_2_, Al_2_O_3_, etc.), and non-metallic materials (glass, PDMS, PTFE, quartz, SiO_2_, etc.) [8].

As is shown in the results of gene expression analysis, IL-10, iNOS, and TNF-α all demonstrated the tendency of suppressing inflammation. It has been previously shown that IL-10 could inhibit the release of inflammatory cytokine and the expression of major histocompatibility complex II, which is the presenter of antigen to T cells [32]. Combining the quantification of both q-PCR and ELISA, we believe the Ti-EUP-Sr could demonstrate promising anti-inflammatory effects. TNF-α, however, is a pro-inflammatory cytokine that has also been comprehensively studied [33], and inducible nitric oxide synthase (iNOS) promotes *in vivo* synthesis of massive nitric oxide (NO), which could be highly pro-inflammatory [34]. The decreased expression of iNOS is especially intriguing, which we believe could be ascribed to the attenuation of the activation of the NF-κB pathway by strontium and the cross-relation between the pro-inflammatory cytokines [35, 36].

The expression of osteogenic genes also demonstrated promising results. OPN has been considered essential in bone formation and was proven to be tightly associated with tooth root development and dental mineralization [37, 38]. OCN is capable of inducing bone formation, modulating bone calcification, and balancing calcium ions [39]. As an early indication of osteoblasts differentiation and proliferation increased ALP results in enhanced bone formation and bone mineralization [40]. Runx2, or runt-related transcription factor 2, is essential in the proper mature process and the differentiation of osteoblasts [41]. With all mentioned genes significantly upregulated in the qPCR assay and the increased expression of OCN further confirmed using ELISA, the alveolar bone regeneration post implantation surgeries would be presumably faster than traditional titanium implants. Our supplementary mineralization assay indeed demonstrated a more prompt and noticeable mineralization (Supplementary Materials S2). Combining with the nanosized porous structure created on the Ti surface, the new bone would anchor into the implant within a reduced healing period. Also, as tissues with abundant vascular system, angiogenesis is another factor contributing to the evaluation of overall recovery time. Interestingly, vascular regenerative genes including VEGFA and EGFL6 are both found upregulated in the qPCR while the increased VEGF expression is identified in ELISA. VEGFA, as an important member of the VEGF protein family, modulates the growth of the vascular endothelium by binding to tyrosine kinase receptors on the plasma membrane, while much attention was paid to EGFL6 in its promotion of endothelial cell migration and angiogenesis via the activation of the extracellular signal-regulated kinase [42, 43].

Focusing on the *in vivo* effects of EUP-Sr-loaded titanium implants, we performed implantation surgeries on rabbits and confirmed the stability of the coating. This is considered essential among contemporary research attempting to modify the surfaces of dental or orthopedic implants, because the mechanical rubbing during the surgery might damage and/or remove the coating, making the modification design completely lost. After confirming that the EUP-Sr coating was maintained after the surgery, as well as its satisfying osseointegration effects, we may anticipate that the polysaccharide complex on the titanium surface will be gradually released to affect its surrounding microenvironments. According to the drug release test conducted in this study, titanium dental implant modified with EUP-Sr can gradually unload the polysaccharide, benefiting the neighboring microenvironment. This design accords with many previously reported surface modification agents for Ti aiming to actively optimize the extracellular microenvironment by gradually releasing certain substances [44–47]. This enables not only the cells and tissues that directly contact the implant but also non-contact neighboring cells to receive the impact of loaded EUP-Sr.

Therefore, we are confident that titanium dental implants modified with EUP-Sr would provide an opportunity to patients with better post-operational performance and a shortened recovery time compared to traditional implants.

## Conclusions

In this work, we proposed a facile approach for the Ti surface modification and employed it to introduce EUP-Sr to Ti surface with the anti-inflammation and osseointegration properties. The successful modification and the great improvement in surface properties were confirmed by combined techniques of SEM/EDS, FTIR, ICP-OES and contact angle measurements. The cell proliferation tests demonstrate that such surface modification was beneficial to improve the cytocompatibility of titanium dental implants. The gene expression analysis reveals that Ti-EUP-Sr displayed positive effects on inflammation-suppression, osteogenesis and angiogenesis. Thus, we confirmed that the approach of modifying titanium surface with polysaccharide complex was valid and that the modification of EUP-Sr to Ti surface could further optimize current dental implants in all three aspects mentioned above. We therefore believed that the Ti-EUP-Sr implant we fabricated here should be promising materials that be used in dental applications and the PDA coating method proposed should be a facile and useful approach to introduce various bioactive contents to Ti surface.

## Electronic supplementary material

Below is the link to the electronic supplementary material.

Supplementary Material 1

Supplementary Material 2

Supplementary Material 3

Supplementary Material 4

Supplementary Material 5

Supplementary Material 6

## Data Availability

All data generated or analyzed during this study are included in this published article.

## List of Abbreviations

Ti: Titanium
Sr: Strontium
EUP: *Eucommia Ulmoides* Polysaccharides
EUP-Sr: Strontium *Eucommia Ulmoides* Polysaccharides
PDA: Polydopamine
Ti-PDA: Titanium modified with polydopamine layer
Ti-EUP-Sr: Titanium modified with Strontium *Eucommia Ulmoides* Polysaccharides (by polydopamine middle layer)
RT: Room temperature
SEM: Scanning Electron Microscopy
EDS: Energy dispersive X-ray spectroscopy
ATR-FTIR: Attenuated total reflection Fourier transform infrared spectroscopy
ICP-OES: Inductively coupled plasma-optical emission spectrometry
HUVECs: Human umbilical vascular endothelial cells
HABOBs: Human alveolar bone osteoblasts
DMEM: Dulbecco’s Modified Eagle Medium
FBS: Fetal bovine serum
RT-qPCR: Real-time quantitative polymerase chain reaction
GAPDH: Glyceraldehyde 3-phosphate dehydrogenase
IL: Interleukin
iNOS: Inducible Nitric Oxide Synthase
TNF: Tumor necrosis factor
ALP: Alkaline phosphatase
OPN: Osteopontin
OCN: Osteocalcin
Runx2: Runt-related transcription factor 2
VEGF: Vascular endothelial growth factor
EGFL6: Epidermal growth factor like-domain 6
ELISA: Enzyme-linked immunosorbent assay
CT: Computer tomography
SD: Standard deviation
