# Supplementary material for: Surface modifications of titanium dental implants with strontium eucommia ulmoides to enhance osseointegration and suppress inflammation
Source: Biomater Res. 2023 Mar 16;27:21. doi: 10.1186/s40824-023-00361-2 (PMC10022180; doi:10.1186/s40824-023-00361-2)
Supplement: Supplementary file 1 — Supplementary Material 1 [file 40824_2023_361_MOESM1_ESM.pdf]

SPRINGER NATURE

Change of authorship request form - Journals (pre-acceptance)

Important information. Please read.

- This form should be used by authors to request any change in authorship (adding/deleting authors) including changes in corresponding authors. This form should not be used for name changes. Please fully complete all sections. Use black ink and block capitals and provide each author's full name with the given name first followed by the family name.

- By signing this declaration, all authors guarantee that the order of the authors are in accordance with their scientific contribution, if applicable as different conventions apply per discipline, and that only authors have been added who made a meaningful contribution to the work.

- Please note, in author collaborations where there is formal agreement for representing the collaboration, it is sufficient for the representative or legal guarantor (usually the corresponding author) to complete and sign the Authorship Change Form on behalf of all authors, **next to the added/removed author(s)**. **(Complete Section 3, followed by Section 6.)**
  In author collaborations where there is no formal agreement for representing the collaboration and **there are more than 10 authors**, one may sign for all, provided the signer appends correspondence that attests that each of the authors have agreed to the change **and the added/removed authors sign the form**. **(Complete Section 3, followed by Section 6.)**

- Please note, we cannot investigate or mediate any authorship disputes. If you are unable to obtain agreement from all authors (including those who you wish to be removed) you must refer the matter to your institution(s) for investigation. Please inform us if you need to do this.

- If you are not able to return a fully completed form within **30 days** of the date that it was sent to the author requesting the change, we may have to withdraw your manuscript. We cannot publish manuscripts where authorship has not been agreed by all authors (including those who have been removed).

- Incomplete forms will be rejected.

- Please return/upload this form, fully completed, to the Journals Editorial Office. The Journal and/or Publisher will consider the information you have provided to decide whether to approve the proposed change in authorship. We may decide to contact your institution for more information or undertake a further investigation, if appropriate, before making a final decision.

SPRINGER NATURE Springer Nature is one of the world's leading global research, educational and professional publishers, created in May 2015 through the combination of Nature Publishing Group, Palgrave Macmillan, Macmillan Education and Springer Science+Business Media.

SPRINGER NATURE

Change of authorship request form - Journals (pre-acceptance)

Section 1: Please provide the current title of manuscript

Manuscript ID no.: BMRS-D-22-00037

Title: Surface Modifications of Titanium Dental Implants with Strontium Eucommia Ulmoides to Enhance Osseointegration and Suppress Inflammation

Section 2: Please provide the previous authorship, in the order shown on the manuscript before the changes were introduced. Please indicate the corresponding author by adding (CA) behind the name.

	First name(s)	Family name	ORCID or SCOPUS id, if available
1st author	Rui	Sun	0000-0002-5991-4435
2nd author	Liqiu	Hu	
3rd author	Yutong	Wu	
4th author	Qili	Sun	
5th author	Xiangchao	Pang	
6th author	Fenbo	Ma	
7th author	Yansong	Wang	
8th author	Jiayi	Liu	
9th author	Bin	Tang (CA)	
10th author			

Please use an additional sheet if there are more than 10 authors.

SPRINGER NATURE Springer Nature is one of the world's leading global research, educational and professional publishers, created in May 2015 through the combination of Nature Publishing Group, Palgrave Macmillan, Macmillan Education and Springer Science+Business Media.

Change of authorship request form - Journals (pre-acceptance)

Section 3: Please provide a justification for change. Please use this section to explain your reasons for changing the authorship of your manuscript, e.g. what necessitated the change in authorship? Please refer to the (journal) policy pages for more information about authorship. Please explain why omitted authors were not originally included and/or why authors were removed on the submitted manuscript.

According to reviewer's comments on supplementing our study with more experiments, we re-allocated some of the revision work to our current postgraduate students Q.S. and Y.W., because the former first author and major contributor to this study, R.S., has joined National University of Singapore and is unable to return to our lab for hands-on experiments due to our local COVID-19 lockdown. Combining their contribution of the initial manuscript preparation, initial rounds of experiments, as well as this round of revision work, we hope to include the three authors, R.S., Q.S., and Y.W., together as first author for their equal contribution.
Another author, X.P., supervised the supplementary experiments during this period together with B.T. who supervised the initial rounds of experiments.Thus, we would like to include both of them as corresponding author with B.T. remains as the main corresponding author.

Section 4: Proposed new authorship. Please provide your new authorship list in the order you would like it to appear on the manuscript. Please indicate the corresponding author by adding (CA) behind the name. <u>If the Corresponding Author has changed, please indicate the reason under section 3</u>.

	First name(s)	Family name (this name will appear in full on the final publication and will be searchable in various abstract and indexing databases)	Affiliated institute	E-mail address
1st author	Rui	Sun	Southern University of Science and Technology	averyrsun@gmail.com
2nd author	Qili	Sun	Southern University of Science and Technology	sunqil1992@126.com
3rd author	Yansong	Wang	Southern University of Science and Technology	12031184@mail.sustech.edu.cn
4th author	Liqiu	Hu	Southern University of Science and Technology	11930748@mail.sustech.edu.cn
5th author	Yutong	Wu	Southern University of Science and Technology	11910731@mail.sustech.edu.cn
6th author	Fenbo	Ma	Southern University of Science and Technology	mafb@mail.sustech.edu.cn
7th author	Jiayi	Liu	Southern University of Science and Technology	11912434@mail.sustech.edu.cn
8th author	Xiangchao	Pang (CA)	Southern University of Science and Technology	pangxiangchao86@163.com
9th author	Bin	Tang (CA)	Southern University of Science and Technology	tangb@sustech.edu.cn
10th author				

Please use an additional sheet if there are more than 10 authors.

SPRINGER NATURE Springer Nature is one of the world's leading global research, educational and professional publishers, created in May 2015 through the combination of Nature Publishing Group, Palgrave Macmillan, Macmillan Education and Springer Science+Business Media.

SPRINGER NATURE

Change of authorship request form - Journals (pre-acceptance)

Section 5: Author contribution, Acknowledgement and Disclosures. Please use this section to provide a new disclosure statement and, if appropriate, acknowledge any contributors who have been removed as authors and ensure you state what contribution any new authors made (if applicable per the journal or book (series) policy). Please ensure these are updated in your manuscript - after approval of the change(s) - as our production department will not transfer the information in this form to your manuscript.

New acknowledgements:

We would like to thank Yixuan Li, a colleague Ph.D. student from Mechanobiology Institute, National University of Singapore, for her help in the processing, reconstruction, and demonstration of micro-CT data.

New Disclosures (financial and non-financial interests, funding):

Not applicable.

New Author Contributions statement (if applicable per the journal policy):

Not applicable.

State 'Not applicable' if there are no new authors.

SPRINGER NATURE Springer Nature is one of the world's leading global research, educational and professional publishers, created in May 2015 through the combination of Nature Publishing Group, Palgrave Macmillan, Macmillan Education and Springer Science+Business Media.

Change of authorship request form - Journals (pre-acceptance)

Section 6: Declaration of agreement. *All* authors, unchanged, new and removed *must* sign this declaration.
(NB: Please print the form, (docu)-sign and return/upload a scanned copy. Please note that signatures that have been inserted as an image file are acceptable as long as it is handwritten. Typed names in the signature box are unacceptable.) *** Please delete as appropriate. Delete all of the bold if you were on the original authorship list and are remaining as an author.**

	First name	Family name		Signature	Date
1st author	Rui	Sun	I agree to the proposed new authorship shown in section 4 and the proposed change in corresponding author	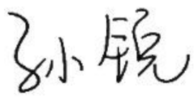	09/24/2022
2nd author	Qili	Sun	I agree to the proposed new authorship shown in section 4 and the proposed change in corresponding author	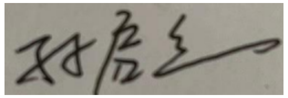	09/24/2022
3rd author	Yansong	Wang	I agree to the proposed new authorship shown in section 4 and the proposed change in corresponding author	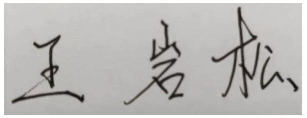	09/24/2022
4th authors	Liqiu	Hu	I agree to the proposed new authorship shown in section 4 and the proposed change in corresponding author	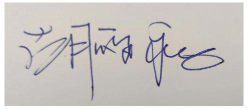	09/24/2022
5th author	Yutong	Wu	I agree to the proposed new authorship shown in section 4 and the proposed change in corresponding author	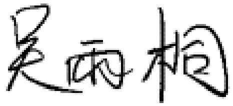	09/24/2022
6th author	Fenbo	Ma	I agree to the proposed new authorship shown in section 4 and the proposed change in corresponding author	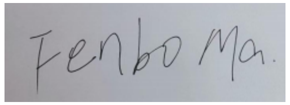	09/24/2022
7th author	Jiayi	Liu	I agree to the proposed new authorship shown in section 4 and the proposed change in corresponding author	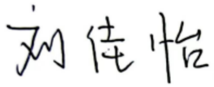	09/24/2022

SPRINGER NATURE Springer Nature is one of the world's leading global research, educational and professional publishers, created in May 2015 through the combination of Nature Publishing Group, Palgrave Macmillan, Macmillan Education and Springer Science+Business Media.

SPRINGER NATURE

Change of authorship request form - Journals (pre-acceptance)

	First name	Family name		Signature	Date
8th author	Xiangchao	Pang	I agree to the proposed new authorship shown in section 4 and the proposed change in corresponding author	庞祥超	09/24/2022
9th author	Bin	Tang	I agree to the proposed new authorship shown in section 4 and the proposed change in corresponding author	Tang [illegible]	09/24/2022
10th author			I agree to the proposed new authorship shown in section 4 /**and the addition/removal*of my name to the authorship list** /and the proposed change in corresponding author		

Please use an additional sheet if there are more than 10 authors.

In case of author collaborations with formal agreement:

	Name of consortium/consortia	First name	Family name		Signature	Date
Representative/legal guarantor				I agree to the proposed new authorship shown in section 4 /**and the addition/removal*of my name to the authorship list** /and the proposed change in corresponding author		

Both added/removed authors should complete the information in the first table under Section 6.

---- End of form ----

SPRINGER NATURE Springer Nature is one of the world's leading global research, educational and professional publishers, created in May 2015 through the combination of Nature Publishing Group, Palgrave Macmillan, Macmillan Education and Springer Science+Business Media.
