# Supplementary material for: Surface modifications of titanium dental implants with strontium eucommia ulmoides to enhance osseointegration and suppress inflammation
Source: Biomater Res. 2023 Mar 16;27:21. doi: 10.1186/s40824-023-00361-2 (PMC10022180; doi:10.1186/s40824-023-00361-2)
Supplement: Supplementary file 2 — Supplementary Material 2 [file 40824_2023_361_MOESM2_ESM.docx]

Supplementary Materials

Surface Modifications of Titanium Dental Implants with Strontium Eucommia Ulmoides to Enhance Osseointegration and Suppress Inflammation

Rui Sun^a, d, e, 1^, Qili Sun^a,1^, Yansong Wang^a,1^, Liqiu Hu^a^, Yutong Wu^a^, Fenbo Ma^a^, Jiayi Liu^a^, Xiangchao Pang^a, f, *^, Bin Tang^a,b,c,*^.

^a^ Department of Biomedical Engineering, Southern University of Science and Technology, Shenzhen, 518055, China

^b^ Guangdong Provincial Key Laboratory of Cell Microenvironment and Disease Research, China

^c^ Shenzhen Key Laboratory of Cell Microenvironment, China

^d^ Department of Biomedical Engineering, College of Design and Engineering, National University of Singapore, Singapore 117583, Singapore

^e^ Mechanobiology Institute (MBI), National University of Singapore, Singapore 117411, Singapore

^f^ College of Materials Science and Engineering, Central South University of Forestry and Technology, Changsha 410004, China

^1^ These authors contributed equally to this study.

*Corresponding author (s)

Main corresponding author: Bin Tang, Email: tangb@sustech.edu.cn; Tel: 86-0755-88018998

**Supplementary material S1**


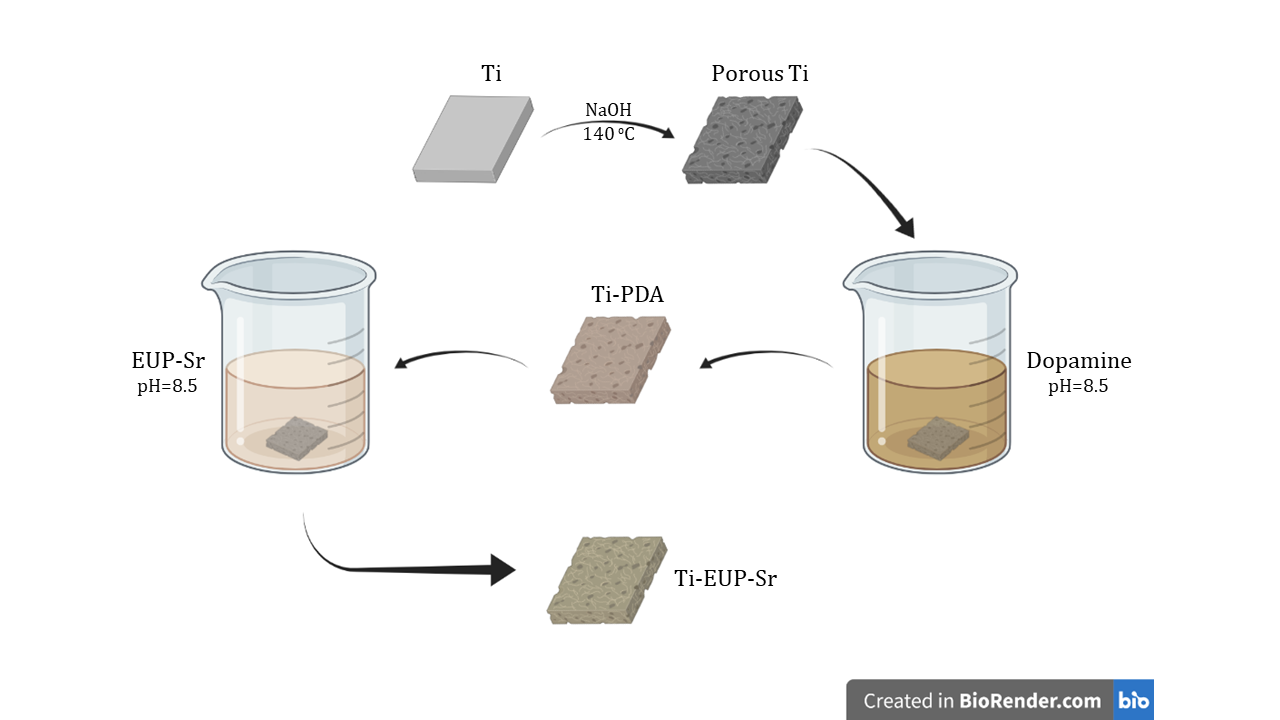


**Supplemental Figure 1.** Schematic illustration of implant surface modified by EUP-Sr. (Created with BioRender.com)
