# Supplementary material for: Surface modifications of titanium dental implants with strontium eucommia ulmoides to enhance osseointegration and suppress inflammation
Source: Biomater Res. 2023 Mar 16;27:21. doi: 10.1186/s40824-023-00361-2 (PMC10022180; doi:10.1186/s40824-023-00361-2)
Supplement: Supplementary file 3 — Supplementary Material 3 [file 40824_2023_361_MOESM3_ESM.docx]

Supplementary Materials

Surface Modifications of Titanium Dental Implants with Strontium Eucommia Ulmoides to Enhance Osseointegration and Suppress Inflammation

Rui Sun^a, d, e, 1^, Qili Sun^a,1^, Yansong Wang^a,1^, Liqiu Hu^a^, Yutong Wu^a^, Fenbo Ma^a^, Jiayi Liu^a^, Xiangchao Pang^a, f, *^, Bin Tang^a,b,c,*^.

^a^ Department of Biomedical Engineering, Southern University of Science and Technology, Shenzhen, 518055, China

^b^ Guangdong Provincial Key Laboratory of Cell Microenvironment and Disease Research, China

^c^ Shenzhen Key Laboratory of Cell Microenvironment, China

^d^ Department of Biomedical Engineering, College of Design and Engineering, National University of Singapore, Singapore 117583, Singapore

^e^ Mechanobiology Institute (MBI), National University of Singapore, Singapore 117411, Singapore

^f^ College of Materials Science and Engineering, Central South University of Forestry and Technology, Changsha 410004, China

^1^ These authors contributed equally to this study.

*Corresponding author (s)

Main corresponding author: Bin Tang, Email: tangb@sustech.edu.cn; Tel: 86-0755-88018998

**Supplementary material S2**

Mineralization assay

Sterilized Ti-EUP-Sr samples and Ti control samples were air dried and placed in a 24 well plate. Simulated body fluid (SBF) was prepared and added to the samples (1 mL/well) to mimic the in vivo electrolytes and osmolarity environment [1]. The well plate was kept on a 100 rpm shaker at 37 ^o^C for 14 days, where the SBF was exchanged every 3 days. The samples were subsequently rinsed by ddH_2_O and kept at 37 ^o^C for 4 hours until fully dried. The samples were then coated with gold and observed with Hitachi Regulus SEM.

As is shown in Fig. S2, mineralized structure was more obviously and ubiquitously identified on the surface of EUP-Sr modified Ti using SEM (Hitachi Regulus). Combining our *in vitro* cell experiments evaluating osteogenic gene expression, we believe Ti-EUP-Sr demonstrates better inducement in bone regeneration.


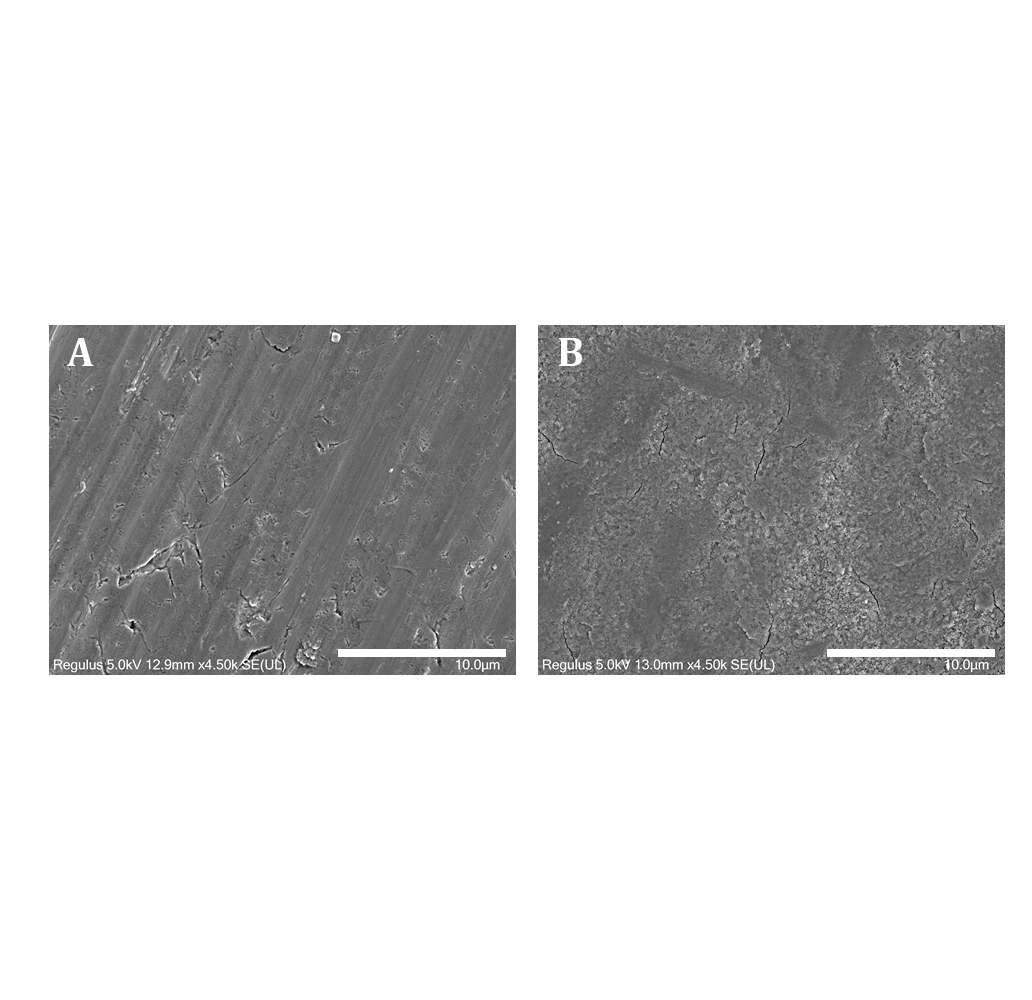


**Fig. S2** EUP-Sr loaded titanium implants (B) demonstrate more effective mineralization compared to untreated Ti (A). Scale bar: 10 μm

Reference

[1] T. Kokubo, H. Kushitani, S. Sakka, T. Kitsugi, and T. Yamamuro, "Solutions able to reproduce in vivo surface-structure changes in bioactive glass-ceramic A-W," (in eng), *J Biomed Mater Res,* vol. 24, no. 6, pp. 721-34, Jun 1990.
