# Supplementary material for: Surface modifications of titanium dental implants with strontium eucommia ulmoides to enhance osseointegration and suppress inflammation
Source: Biomater Res. 2023 Mar 16;27:21. doi: 10.1186/s40824-023-00361-2 (PMC10022180; doi:10.1186/s40824-023-00361-2)
Supplement: Supplementary file 6 — Supplementary Material 6 [file 40824_2023_361_MOESM6_ESM.docx]

Dear Editor:

We sincerely appreciate the opportunity to revise our manuscript (BMRS-D-22-00037) entitled: “Surface Modifications of Titanium Dental Implants with Strontium Eucommia Ulmoides to Enhance Osseointegration and Suppress Inflammation”. We have carefully considered all the comments and revised the paper accordingly.

We found the comments from the reviewers really insightful and helpful in making our work more comprehensive and conclusions more persuasive. Herein, we explained how we designed supplementary experiments and revised the manuscript based on reviewer’s comments and suggestions. For your convenience, the revised parts in the manuscript are highlighted in yellow. Hopefully, all these revisions could elucidate the concerns addressed by the reviewers.

According to reviewer's comments on supplementing our study with more experiments, we re-allocated some of the revision work to our current postgraduate students Q.S. and Y.W., including sample preparations and most of the *in vitro* and *in vivo* experiments. Another author, X.P., supervised the supplementary experiments during this period together with B.T. who supervised the initial rounds of experiments. Thus, we would like to include all of R.S. (initial first author), Q.S., and Y.W. as first authors due to their equal contribution in the entire process, while X.P. and B.T. both are included as corresponding authors. All authors have agreed to the mentioned changes, with their consent signature and more details addressed in the attached form.

We would like to appreciate the kind help from the editorial office during our revision process, and we hope this revision can be considered qualified for acceptance.

Looking forward to hearing from you soon.

Best regards,

Bin Tang

--------------------------------------------------

Dr. Bin Tang

Department of Biomedical Engineering

Southern University of Science and Technology

Shenzhen 518055, P. R. China

Tel/Fax: +86-755-88018923

E-mail: tangb@sustc.edu.cn

**Reply to Reviewer #1:**

1. ***In introduction, the biological effect of EUP-Sr should be described in more detail.***

The authors would like to thank you for this comment. We agree that with more detailed elaborations on the biological effect of EUP-Sr, the potential readers would obtain more necessary background knowledge of how EUP-Sr is actively regulating the osteoimmune system. We did further literature research on the biological effects of EUP-Sr and include more details in the introduction part regarding this topic.

**In 1. Introduction:** “Combined with strontium acting as an osteoblastogenesis initiator, strontium *Eucommia Ulmoides* polysaccharides (EUP-Sr) were synthesized and investigated in a previous work. The polysaccharide complex demonstrated no cytotoxicity in the cell proliferation test, while significantly inhibited multiple proinflammatory factors in the gene expression of macrophages. It was also proven that EUP-Sr could potentially induce bone regeneration by upregulating osteoblastogenesis gene expression while suppressing osteoclastogenesis.”

**In 1.** **Introduction:** “In vivo assays confirmed that the EUP-Sr loaded onto the implant could endure surgical manipulations when the surface was rubbed with the alveolar bone, while the polysaccharide complex can actively and consistently benefit the neighboring microenvironment in the recovery process.”

2. ***What is chemical structure of EUP, and how do EUP and Sr bind each other?***

We appreciate this comment. EUP is a natural polysaccharide extracted from a traditional Chinese herbal medicine, *Eucommia Ulmoides Oliv*. As a bio-macromolecule, the higher structure of EUP is rather complicated (similar idea as the 3-D folding of different proteins). But researchers have managed to understand the primary structure of many polysaccharides including EUP, which can potentially build a fundamental understanding of their higher structures [1]. As other natural carbohydrate polymers originated from plants (e.g., *Astragalus* polysaccharides [2], *Achyranthes bidentata* polysaccharides [3], *Lycium barbarum* polysaccharides [4], etc.), EUP possess such a complicated molecular structure that current chemical characterizations mainly follow the general protocol and criteria by first analyzing the monosaccharide composition, then investigating how they are interconnected to generate a macromolecule [5,6]. To be specific, gas chromatography revealed that EUP contains 36.6% (mass, so are the followings) glucose, 16.6% fructose, 15.7% fucose, 14.2% mannose, 9.5% galactose, and 7.4% arabinose [5]. Further analyzing the FTIR spectra reveals that apart from the ubiquitous C-H and C=O attachments within/between monosaccharides, β-type glycosidic bonds and hydrogen bonds are also the contributors to the primary structure of EUP in the aspect of monosaccharide interconnection [5,6]. Based on these generally acknowledged information about EUP chemical structure, a previous study sought to modify EUP with strontium attachment for the first time [6]. Identifying the intensity change of the peak at 3424 cm^-1^ and peak shifting at 1000 cm^-1^ in the FTIR spectra, it is believed that Sr incorporations are ascribed to hydrogen bonds. The ^1^H NMR spectra further confirmed their hypotheses, revealing the contributions of hydroxyl groups and glycosidic linkages in creating EUP-strontium linkage.

3. ***In introduction or discussion, authors should describe or speculate the interaction between EUP-Sr and PDA in terms of chemical interaction.***

The authors appreciate this helpful comment because by adding a description or speculation of the mechanism to attach EUP-Sr to PDA layer, the general flow and logic connections between certain subsections can be further improved. We have added a speculative statement in the introduction part, while the verification of our anticipation, as the original manuscript was organized, is shown in 3.1.2 based on the ATR-FTIR results.

**In 1. Introduction:** “It was proven that PDA-modified surfaces can be further coated with a secondary drug/chemical by creating non-covalent linkages including hydrogen bonds, π stacking, and van der Waals interaction. Thus, it can be speculated that the ubiquitous existence, as well as the hydrogen-bond-availability of glycosidic linkages in various types of polysaccharide complexes, enables the introduction of a secondary polysaccharide coating onto the PDA layer.”

4. ***Fig.1 may be not needed in this manuscript.***

The initial consideration of the authors to include this figure is to provide a direct and illustrative workflow of Ti-EUP-Sr synthesis as an easy reference for potential readers. But according to your comment, we have moved this picture to the supplementary material (Supplementary Materials S1).

5. ***In Fig. 6 and 7, what kind of control was used? Was it well plate, uncoated Ti, or PDA-Ti? It might be ideal that both uncoated Ti and PDA-Ti are used as controls.***

The authors apologize for our ambiguity in explaining our control group. In Fig. 5 and 6 in the revised manuscript (former Fig. 6 and 7. Indexes were changed according to your comment #4), the control groups used untreated Ti representing traditional titanium dental implants. Corresponding explanations are now included in subsections 3.2.1 and 3.2.2, which could hopefully eliminate any possible confusions.

Your suggestion regarding using both uncoated Ti and Ti-PDA as control groups for our *in vitro* study is totally understandable because it will provide more comprehensive and convincing controls towards different chemicals that we used. However, the authors would like to respectfully argue that using both Ti and Ti-PDA as controls may not be necessary because:

(1) The main focus of the cell proliferation assay and qPCR test is to compare the biocompatibility, cytotoxicity, and active biological functions of our overall product (i.e. Ti-EUP-Sr) with current dental implants (i.e. untreated Ti). Therefore, we believe by using untreated Ti, our hypotheses can be validated that Ti-EUP-Sr demonstrates no cytotoxicity while potentially provides better effects in suppressing inflammation and promoting tissue repair (osseointegration and angiogenesis) than traditional Ti dental implants after implantation surgery.
(2) The biological influence of PDA surface modification has been investigated thoroughly after its first report in 2007 [7]. It has been previously proved that PDA coating is either mildly toxic or non-toxic to cells [8–10], favorable for cell attachment [11], while Ti-PDA is likely to inhibit the expression of osteoblastic gene expression [10]. Thus, we may intuitively assume that, to apply PDA treatment in the field of bone tissue engineering, the “secondary drug” loaded on PDA layer (in our study, EUP-Sr) should possess satisfying ability in inducing osteointegration and regeneration to counter this inhibition for bone regeneration. This is achieved not only by the promising effects of EUP-Sr, but also another intriguing property of PDA coating that has also been studied: its ability in promoting drug uptake [8,12], which can potentially further enhance the biological effects of the “secondary drug”.

Therefore, guided by our main focus in this study, based on previous studies for PDA toxicity and biological effects, and the fact that many studies using PDA as a coating layer no longer include substrate-PDA as one of the control groups [12,13], the authors would like to respectfully keep our control group settings as it is (untreated Ti).

6. ***Only RT-PCT results are too limited for demonstration of immuno-regulatory and osteogenic effects. ELISA and mineralization assay are needed at least.***

The authors agree that by supplementing our study with ELISA and mineralization assay would provide more persuasive and comprehensive evaluations for our modified titanium implants. Key genes expression related to immune-regulation (IL-10), osteogenesis (OCN), and angiogenesis (VEGF) were evaluated using ELISA. A 14-days mineralization assay is now included in the supplementary materials S2. We appreciate this suggestion and we hope these results can resolve your concerns.

**(ELISA Methods) In 2.5.4 Enzyme-linked immunosorbent assay (ELISA):** “The ELISA assay kits used in this study (human VEGF ELISA kit, human OCN ELISA kit, and mouse IL-10 ELISA kit) were bought from Beyotime Biotechnology. Assays were conducted according to supplier’s instructions came with each kit. Briefly, after culturing RAW264.7, HUVECs, and HABOBs for 1, 3, or 7 days on EUP-Sr modified Ti samples, cell supernatant was obtained and diluted before adding 100 μL samples from each group to a 96 well plate. Standard curves (2000, 1000, 500, 250, 125, 62.5 pg/mL) were obtained with every assay proceeded. After adding biotinylated antibodies, streptavidin labeling solution, and TMB solution stepwisely according to the product instruction (5 times of washing are required before adding each solutions mentioned), the well plates were incubated in dark for 20 minutes and supplemented with 50μL/well termination solution. A450 values were measured using a microplate reader (iMark, Bio-Rad).”

**(ELISA Results) In 3.2.3 ELISA:** “To provide a down-stream evaluation of gene expressions of the mentioned cells in response to EUP-Sr loaded titanium implants, ELISA targeting at vascular endothelial growth factor (VEGF), OCN, and IL-10 were conducted using HUVECs, HABOBs, and RAW264.7 cells, respectively. As shown in Fig. 7, the downstream expressions of IL-10 in RAW264.7 cells and VEGF in HUVECs were both immediately upregulated within only 24 hours of culture. After 3 days, the OCN expression in HABOBs and VEGF expression in HUVECs showed a significant increase. These results further confirmed the osteo-immunoregulatory functions of Ti-EUP-Sr, as well as its promising angiogenetic inducement.”

**Fig. 7.** ELISA results confirm increased angiogenesis and osteo-immunoregulatory function.

**(Mineralization assay methods & results) In Supplementary Materials S2:** “Sterilized Ti-EUP-Sr samples and Ti control samples were air dried and placed in a 24 well plate. Simulated body fluid (SBF) was prepared and added to the samples (1 mL/well) to mimic the in vivo electrolytes and osmolarity environment [1]. The well plate was kept on a 100 rpm shaker at 37 oC for 14 days, where the SBF was exchanged every 3 days. The samples were subsequently rinsed by ddH2O and kept at 37 oC for 4 hours until fully dried. The samples were then coated with gold and observed with Hitachi Regulus SEM.

As is shown in Fig. S2, mineralized structure was more obviously and ubiquitously identified on the surface of EUP-Sr modified Ti using SEM (Hitachi Regulus). Combining our in vitro cell experiments evaluating osteogenic gene expression, we believe Ti-EUP-Sr demonstrates better inducement in bone regeneration.”

**Fig. S2** EUP-Sr loaded titanium implants (B) demonstrate more effective mineralization compared to untreated Ti (A). Scale bar: 10 μm

**Reply to Reviewer #2:**

1. ***3.1.1. Morphology of samples: It is needed to add direct experiments to support the immobilization. Increasing mass is possible with simple adsorption, which must be distinguished from immobilization. SEM observation is recommended by cutting and showing the sample cross-section or scratching the surface with physical force.***

The authors are sorry if we failed to elaborate this in the original manuscript. We agree that showing the SEM cross-sectional view by cutting or scratching the sample would provide a more quick-and-direct confirmation of the immobilization. The mentioned figure is now included in the revised manuscript showing the coating on titanium surface by physical cutting. We agree that adsorption and immobilization can both increase the mass indeed, but we have also confirmed that the EUP-Sr is chemically immobilized onto Ti-PDA surface by forming hydrogen bonds between the glycosidic linkage of EUP-Sr and the ring structure of PDA, according to the peak shifting in the FTIR spectra (section 3.1.2). This phenomenon has also been noticed and fully addressed in a previous study [14]. We sincerely appreciate this kind suggestion and hope the revised manuscript can address this necessary question properly.

**(SEM method) In 2.4.1 SEM and SEM/EDS:** “To directly confirm and visualize the EUP-Sr coating, the sample was cut and the cross-sectional surface was observed with SEM.”

**(SEM result) In 3.1.1 Morphology of samples:** “Subsequently, we observed the cross-sectional surface of both Ti (Fig. 1E) and Ti-EUP-Sr (Fig. 1F) with 24 h treatment time using SEM, finding a uniform layer of EUP-Sr coating.”

**Fig. 1.** SEM images of hydrothermal alkaline treated Ti (A), Ti-PDA (B), Ti-EUP-Sr-12H (C), and Ti-EUP-Sr-24H (D). **Cross sectional view of Ti (E) and Ti-EUP-Sr-24H (F)**. EDS result of strontium content (G). In Fig. 1(G), values shown are mean ± SD. ⁎⁎p<0.01 or ⁎⁎⁎⁎p<0.0001 stands for a significant difference (no Sr signals were detected in the Ti group).

2. ***Real-time PCR for only a few genes at a time point makes it difficult to conclude anti-inflammation, osteogenic potential, and angiogenesis functions. It is necessary to analyze the gene expression pattern with time series, not just one time. In addition, more molecular work is needed to support the conclusion, anti-inflammation, osteogenic potential, and angiogenesis functions of Sr.***

The authors would like to thank you for this comment. We agree that harvesting cells for assays at different time points would make the result more conclusive, and including other in vitro assays such as ELISA and mineralization assay would make our results more comprehensive. As suggested, the RT-qPCR now includes the results after 1 and 3 days (RAW264.7 cells and HUVECs) or after 3 and 7 days (HABOBs). We also performed ELISA assays targeting at essential genes for immuno-regulation (IL-10), osteo-regeneration (OCN), and angiogenesis (VEGF), as well as a mineralization assay (in supplementary materials S2) to better evaluate the Ti-EUP-Sr.

**(ELISA Methods) In 2.5.4 Enzyme-linked immunosorbent assay (ELISA):** “The ELISA assay kits used in this study (human VEGF ELISA kit, human OCN ELISA kit, and mouse IL-10 ELISA kit) were bought from Beyotime Biotechnology. Assays were conducted according to supplier’s instructions came with each kit. Briefly, after culturing RAW264.7, HUVECs, and HABOBs for 1, 3, or 7 days on EUP-Sr modified Ti samples, cell supernatant was obtained and diluted before adding 100 μL samples from each group to a 96 well plate. Standard curves (2000, 1000, 500, 250, 125, 62.5 pg/mL) were obtained with every assay proceeded. After adding biotinylated antibodies, streptavidin labeling solution, and TMB solution stepwisely according to the product instruction (5 times of washing are required before adding each solutions mentioned), the well plates were incubated in dark for 20 minutes and supplemented with 50μL/well termination solution. A450 values were measured using a microplate reader (iMark, Bio-Rad).”

**(ELISA Results) In 3.2.3 ELISA:** “To provide a down-stream evaluation of gene expressions of the mentioned cells in response to EUP-Sr loaded titanium implants, ELISA targeting at vascular endothelial growth factor (VEGF), OCN, and IL-10 were conducted using HUVECs, HABOBs, and RAW264.7 cells, respectively. As shown in Fig. 7, the downstream expressions of IL-10 in RAW264.7 cells and VEGF in HUVECs were both immediately upregulated within only 24 hours of culture. After 3 days, the OCN expression in HABOBs and VEGF expression in HUVECs showed a significant increase. These results further confirmed the osteo-immunoregulatory functions of Ti-EUP-Sr, as well as its promising angiogenetic inducement.”

**Fig. 7.** ELISA results confirm increased angiogenesis and osteo-immunoregulatory function.

**(Mineralization assay methods & results) In Supplementary Materials S2:** “Sterilized Ti-EUP-Sr samples and Ti control samples were air dried and placed in a 24 well plate. Simulated body fluid (SBF) was prepared and added to the samples (1 mL/well) to mimic the in vivo electrolytes and osmolarity environment [1]. The well plate was kept on a 100 rpm shaker at 37 oC for 14 days, where the SBF was exchanged every 3 days. The samples were subsequently rinsed by ddH2O and kept at 37 oC for 4 hours until fully dried. The samples were then coated with gold and observed with Hitachi Regulus SEM.

As is shown in Fig. S2, mineralized structure was more obviously and ubiquitously identified on the surface of EUP-Sr modified Ti using SEM (Hitachi Regulus). Combining our in vitro cell experiments evaluating osteogenic gene expression, we believe Ti-EUP-Sr demonstrates better inducement in bone regeneration.”

**Fig. S2** EUP-Sr loaded titanium implants (B) demonstrate more effective mineralization compared to untreated Ti (A). Scale bar: 10 μm

3. ***There are many similar studies to improve the osseointegration via surface modification or coating, and there is a high possibility that coating materials like as Sr will be detected around and lost because dental implant is clinically rubbed with the alveolar bone during implantation. Further experiments are needed on this.***

We sincerely appreciate this considerate suggestion based on your concern connecting our study with actual clinical situations. We agree that we haven’t realized this aspect before and we are more than happy to clarify this concern with further experiments. Now we include an *in vivo* study in our revised manuscript. EUP-Sr coated titanium implants (the implant body is a commercially available product in dentistry) were inserted to rabbits using the same technique as human dental implantation surgery (the animal surgery was done by a locally-prestigious dentist), and hopefully the results can attenuate this necessary concern.

**(Surgery procedure) In section 2.6 Animal experiments:** “The in vivo experiment of this study was approved and conducted in Southern University of Science and Technology (Approval#: SCXK2022-0170), where New Zealand rabbits (female, 85-115 days, 2-2.5 kg) were purchased from Guangdong Medical Laboratory Animal Center. The animals were restrained and anesthetized using isoflurane with an increasing concentration gradient, followed by hair removal and iodine sterilization at the surgical area (back right tibia). 2% of lidocaine was used at the surgical area to enhance regional anesthesia followed by further iodine sterilization and a ~5 cm longitudinal incision until the upper-middle region of the tibia was exposed. Periosteum and deep muscular tissue were identified and carefully dragged towards the side to expose the tibia surface. Pre-cooled (4 oC) saline was used to rinse the implantation region, and a 0.5 mm round bur was used to create a ~0.5 mm × 0.5 mm locating region on the cortical bone. Commercialized Ti implants or EUP-Sr modified Ti implants were then inserted through the locating region while screwing. The surgical region was then closed and the animal was recovered. After euthanasia, implants were removed from the tibiae after bone extraction followed by SEM/EDS analysis of the implantation region. The tibiae of the other animals were extracted for micro-CT imaging and reconstructed using Imaris software.”

**(Results) In section 3.3 *In vivo* evaluations of Ti-EUP-Sr:** “After the implantation surgery, we (1) immediately sacrificed the animal, harvest the surgical region of the tibia, and removed the inserted implants for SEM/EDX tests, and also (2) performed micro-CT on the experimental animal to confirm the osseointegration. Fig. 8A-D shows the key surgical procedures including tibia exposure, implantation, suturing, sample extraction after euthanasia. From a sample image of the micro-CT reconstruction shown as Fig. 8E/F (re-slicing animation of the entire sample can be seen at Supplementary Materials S3_CT_Control and Supplementary Materials S4_CT_Ti-EUP-Sr), the implants also demonstrated satisfying osseointegration after surgery. In Fig. 8G, negligible amount of strontium was detected around the implantation region, indicating that the EUP-Sr coating remained stable even after the mechanical disturbance during the surgical rubbing between the implant surface and tibia.”

**F Fig. 8.** The key surgical procedures and results for *in vivo* experiments. Tibia exposure (A), implantation (B), closure and recover (C), and sample extraction post-euthanasia (D). Micro-CT reconstruction demonstrated satisfying osteointegration of the implant (control: E; Ti-EUP-Sr: F), while the SEM/EDS results confirmed the coating stability with negligible strontium detected around the surgical area (G).

**(Discussion) In section 4. Discussion:** “Focusing on the in vivo effects of EUP-Sr-loaded titanium implants, we performed implantation surgeries on rabbits and confirmed the stability of the coating. This is considered essential among contemporary research attempting to modify the surfaces of dental or orthopedic implants, because the mechanical rubbing during the surgery might damage and/or remove the coating, making the modification design completely lost. After confirming that the EUP-Sr coating was maintained after the surgery, as well as its satisfying osseointegration effects, we may anticipate that the polysaccharide complex on the titanium surface will be gradually released to affect its surrounding microenvironments.”

***Minor comment: 1. Please use a unified word; osteointegration/osseointegration***

We appreciate your carefulness in reading and evaluating our manuscript. The revised manuscript is now using only the word “osseointegration”. We sincerely apologize for any confusion caused.

**Reference**

[1] P. Zeng, J. Li, Y. Chen, L. Zhang, The structures and biological functions of polysaccharides from traditional Chinese herbs, Prog Mol Biol Transl. 163 (2019) 423–444. https://doi.org/10.1016/bs.pmbts.2019.03.003.

[2] Y. Zheng, W. Ren, L. Zhang, Y. Zhang, D. Liu, Y. Liu, A Review of the Pharmacological Action of Astragalus Polysaccharide, Front Pharmacol. 11 (2020) 349. https://doi.org/10.3389/fphar.2020.00349.

[3] Z. Lin, T. Li, Q. Yu, H. Chen, D. Zhou, N. Li, C. Yan, Structural characterization and in vitro osteogenic activity of ABPB-4, a heteropolysaccharide from the rhizome of Achyranthes bidentata, Carbohyd Polym. 259 (2021) 117553. https://doi.org/10.1016/j.carbpol.2020.117553.

[4] C.C. Wang, S.C. Chang, B.H. Chen, Chromatographic determination of polysaccharides in Lycium barbarum Linnaeus, Food Chem. 116 (2009) 595–603. https://doi.org/10.1016/j.foodchem.2009.03.015.

[5] H. Feng, J. Fan, Z. Song, X. Du, Y. Chen, J. Wang, G. Song, Characterization and immunoenhancement activities of Eucommia ulmoides polysaccharides, Carbohydr Polym. 136 (2016) 803–811. https://doi.org/10.1016/j.carbpol.2015.09.079.

[6] Y. Deng, F. Ma, L.I. Ruiz-Ortega, Y. Peng, Y. Tian, W. He, B. Tang, Fabrication of strontium Eucommia ulmoides polysaccharides and in vitro evaluation of their osteoimmunomodulatory property, Int J Biol Macromol. 140 (2019) 727–735. https://doi.org/10.1016/j.ijbiomac.2019.08.145.

[7] H. Lee, S.M. Dellatore, W.M. Miller, P.B. Messersmith, Mussel-Inspired Surface Chemistry for Multifunctional Coatings, Science. 318 (2007) 426–430. https://doi.org/10.1126/science.1147241.

[8] X. Liu, J. Cao, H. Li, J. Li, Q. Jin, K. Ren, J. Ji, Mussel-Inspired Polydopamine: A Biocompatible and Ultrastable Coating for Nanoparticles in Vivo, ACS Nano. 7 (2013) 9384–9395. https://doi.org/10.1021/nn404117j.

[9] W. Chen, M. Qin, X. Chen, Q. Wang, Z. Zhang, X. Sun, Combining photothermal therapy and immunotherapy against melanoma by polydopamine-coated Al2O3 nanoparticles, Theranostics. 8 (2018) 2229–2241. https://doi.org/10.7150/thno.24073.

[10] T. Shen, W. Yang, X. Shen, W. Chen, B. Tao, X. Yang, J. Yuan, P. Liu, K. Cai, Polydopamine-Assisted Hydroxyapatite and Lactoferrin Multilayer on Titanium for Regulating Bone Balance and Enhancing Antibacterial Property, Acs Biomater Sci Eng. 4 (2018) 3211–3223. https://doi.org/10.1021/acsbiomaterials.8b00791.

[11] Y. Hu, W. Dan, S. Xiong, Y. Kang, A. Dhinakar, J. Wu, Z. Gu, Development of collagen/polydopamine complexed matrix as mechanically enhanced and highly biocompatible semi-natural tissue engineering scaffold, Acta Biomater. 47 (2017) 135–148. https://doi.org/10.1016/j.actbio.2016.10.017.

[12] X. Chen, R. Tong, B. Liu, H. Liu, X. Feng, S. Ding, Q. Lei, G. Tang, J. Wu, W. Fang, Duo of (–)-epigallocatechin-3-gallate and doxorubicin loaded by polydopamine coating ZIF-8 in the regulation of autophagy for chemo-photothermal synergistic therapy, Biomater Sci-Uk. 8 (2019) 1380–1393. https://doi.org/10.1039/c9bm01614g.

[13] Z. Tapsir, F.H. Jamaludin, B. Pingguan-Murphy, S. Saidin, Immobilisation of hydroxyapatite-collagen on polydopamine grafted stainless steel 316L: Coating adhesion and in vitro cells evaluation, J Biomater Appl. 32 (2018) 987–995. https://doi.org/10.1177/0885328217744081.

[14] O. Pop-Georgievski, D. Kubies, J. Zemek, N. Neykova, R. Demianchuk, E.M. Chánová, M. Šlouf, M. Houska, F. Rypáček, Self-assembled anchor layers/polysaccharide coatings on titanium surfaces: a study of functionalization and stability, Beilstein J Nanotechnol. 6 (2015) 617–631. https://doi.org/10.3762/bjnano.6.63.
